
JOURNAL INFORMATION
==============================
NLM Title Abbreviation: J Health Pollut
Journal ID: J Health Pollut
NLM Journal ID: 101690849
Journal ID: hapn

Journal of Health & Pollution

EISSN: 2156-9614
Publisher: National Institute of Environmental Health Sciences

ARTICLE INFORMATION
==============================
PMCID: PMC6221435
DOI: 10.5696/2156-9614.8.17.1
Article version: 1
Subjects: Supplement

Proceedings of the 2nd International DiMoPEx conference

Abstracts Conference proceedings

Publication date: 2018 Mar
Electronic publication date: 2018 Mar 12
Volume: 8
Issue: 17
First page: S1
Last page: S77
Copyright: © 2018 Pure Earth
Copyright year: 2018
License: This is an Open Access article distributed under the terms of the Creative Commons Attribution Licence (http://creativecommons.org/licenses/by/3.0), which permits unrestricted use, distribution and reproduction in any medium, provided the original author and source are credited.
License URL: https://creativecommons.org/licenses/by/3.0/

Page count: 77

==============================
### 2nd INTERNATIONAL CONFERENCE

### DiMoPEx Working Groups Meeting

### Pollution in Living and Working Environments and Health

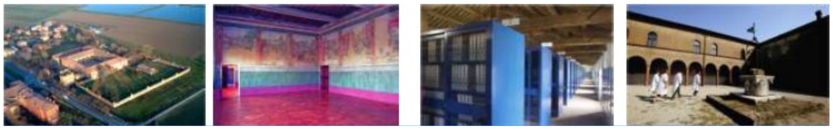

### Abstracts

### Conference proceedings

All sessions presented at the 2nd International DiMoPEx Conference on
"Pollution in Living and Working Environments and Health,"
DiMoPEx Working Groups Meeting

**30–31 October 2017**

Cesare Maltoni Cancer Research Center
Ramazzini Institute, Bentivoglio, near Bologna, Italy

PRESENTED BY

Multicenter EU COST project, CA 15129 DiMoPEx (Diagnosis, Monitoring and Prevention of Exposure-related Non-communicable Diseases)

ENDORSED BY

Collegium Ramazzini

J|H&P
JOURNAL OF HEALTH AND POLLUTION

BERNARDINUS · RAMAZZINUS · PROF. & MEDICINÆ · DOCTOR · ÆTATIS · LXXXI

DiMoPEx

### Editorial

Lygia Therese Budnik (1), Fiorella Belpoggi (2), Ludwine Casteleyn (3)

(1) University Medical Center Hamburg-Eppendorf, Institute for Occupational and Maritime Medicine (ZfAM), Translational Toxicology and Immunology Unit, Hamburg, Germany
(2) Ramazzini Institute, Cesare Maltoni Cancer Research Center, Bentivoglio, Bologna, Italy
(3) University of Leuven, Center for Human Genetics and Center for Environment and Health, Leuven, Belgium

Action Chair:
Lygia Therese Budnik
l.budnik@uke.de

Local Organizer:
Fiorella Belpoggi
belpoggif@ramazzini.it

Action Vice Chair:
Ludwine Casteleyn
Ludwine.Casteleyn@kuleuven.be

### Overview

The WHO has ranked environmental exposures among the top risk factors for chronic disease mortality. Worldwide, about 55 million people die each year from noncommunicable diseases (NCDs) including cancer, diabetes, and chronic cardiovascular, neurological and lung diseases. External exposures in living and working environments alongside with individual susceptibility and lifestyle-driven factors contribute to NCD etiologies.[1] Research addressing the links between environmental exposures and disease prevalence is key for preventing of the pandemic increase in NCD morbidity and mortality. However, because of their long latency and chronic course of some diseases and the necessity to address cumulative exposures over very long periods, it is often difficult to identify causal environmental exposures.[1]

EU-funded COST Action DiMoPEx (Diagnosis, Monitoring, and Prevention of Exposure-related Non-communicable Diseases) develops new concepts for a better understanding of health-environment (including gene-environment) interactions in the etiology of NCDs. The overarching idea is to teach and train scientists and physicians how to include efficient and valid exposure assessments in research and clinical settings in current and future cooperative projects.[2] DiMoPEx partners have identified some of the emerging research needs, which include the lack of evidence-based exposure data, the need for human-equivalent animal models mirroring the human lifespan and low-dose cumulative exposures. Utilizing an interdisciplinary approach, including seven working groups, DiMoPEx focuses on aspects of air pollution with particulate matter including dust and fibers, exposures to low-dose solvents and sensitizing agents. Biomarkers of early exposures and the associated effects as indicators of disease-derived information will be tested and standardized within individual projects. Risks of some NCDs, such as pneumoconioses and some cancers and allergies, are predictable and preventable. Consequently, preventive actions could lead to decreasing disease morbidity and mortality for many of the NCDs of major public concern. DiMoPEx plans to catalyze and stimulate interaction of scientists with policy-makers on exposure-related diseases of concern.

### The DiMoPEx Organization

The predominant goal is to help scientists, physicians, and health officials to prevent and reduce health impacts associated with various exposure scenarios. Seven working groups (WG1–WG7) address the tools to include evidence-based exposure assessment (in research and clinical settings), using modern methods such as ambient monitoring and human biomonitoring methods (WG1, WG2), attached with various biomarkers of effect and susceptibility alongside the clinical diagnostic methods and biomarker-based evaluation of lifestyle factors (WG3, WG6). This is expected to result in the development of cooperative projects (WG7), which cannot be covered as a whole by the individual disciplines (i.e. epidemiology or the traditional environmental medicine only).[1,2]

### The Objectives of the Conference

DiMoPEx aims to foster capacity-building and bring together scientists, clinical researchers and practitioners in the relevant (sub-) disciplines. It offers a platform for interdisciplinary capacity-building between researchers across Europe. DiMoPEx aims also to attract and focus the interests of the next generation of early career investigators on key emerging issues of exposure-related disease burden and various aspects of exposure assessment sciences.[3] The separate sessions will focus on the current status in the field, future needs, and special methods and tools needed to reach the project goals.[1]

### The Scientific Program

The scientific program of the 2nd DiMoPEx Conference includes lectures, short oral presentations and posters presented by scientists from 26 countries covering a broad spectrum of topics. The topics are related to the topics of the 7 DiMoPEx working groups. WG 1 (Exposure assessment), WG 2 (Toxicology, Management and Risk Assessment of Chemicals), WG 3 (Environmental and Occupational Epidemiology), WG 4 (Ethical, Social and Legal Aspects of Risk Communication), WG 5 (Genotoxicity and Susceptibility), WG 6 (Burden of Noncommunicable Diseases and Clinical Diagnosis), WG 7 (Dissemination and Implementation of New Knowledge) and also to joint cooperative projects between the working groups and/or external project partners.[2]
Assessment of the impacts of air pollution on population health and the evaluation of trends relative to other risk factors requires accurate exposure data from living and working environments[1,2] and is within the upcoming focus of all DiMoPEx working groups.
Exposure to air pollution is a major risk factor for global disease.[4] Professor Philip Landrigan clearly demonstrated in his plenary lecture that ambient air, chemical and soil pollution are increasing, and non-communicable diseases (NCDs) caused by these modern forms of pollution are on the rise. Pollution-related diseases were responsible for 9 million premature deaths in 2015—three times as many deaths as AIDS, tuberculosis and malaria combined.[5]

Within several joint research projects, DiMoPEx partners are focusing on the impact of pollution on human health. The projects concentrate on several pollutants (particulate mass fractions PM2.5 and PM10, and a range of metals, inorganic gases and organic compounds) in living and working environments. High contributions occur from industrial point sources in urban air due primarily to road traffic, residential wood combustion, energy production and in rural areas due to agricultural activities, demonstrating the association of nitrate exposures in drinking water and colorectal cancer or atmospheric ammonia exposure to preschool children (Sigsgaard and colleagues), for example. A recent epidemiological study investigates the relationship between air quality and risk of non-communicable diseases in Belgium (Guilbert et al.). Other projects address the health effects of pollution such as mixed dust pneumoconiosis (Baur et al.) and the effects of indoor air pollutants on chronic respiratory diseases (Goeksel et al.). Gustavsson and colleagues showed an increased risk for children to be born small for gestational age (low birth weight or preterm) if their mothers are exposed to welding fumes during pregnancy (abstract not shown here) and Hansen et al. observed a deterioration in neurocognitive performance after chronic pyrethroid exposure. Several groups of DiMoPEx project partners have developed methods for other project partner (i.e. Pelit et al., Göen et al., and Pulver et al.) and demonstrated their application. The micronucleus (MN) frequency biomonitoring approach has been recognized as a promising tool, especially among populations exposed to multiple carcinogens simultaneously (Bolognesi et al.; Teixeira and coworkers). A biomarker-based approach using MN frequencies in nasal and buccal cells has also been proposed to monitor workers exposed to aero-digestive carcinogens to reduce the occurrence of occupational cancer (Hopf et al.). As a part of the pilot study on glyphosate/Roundup toxicity, ongoing at the Cesare Maltoni Cancer Research Center, the frequency of MN was evaluated in the bone marrow of rats treated from in the embryonic stage at the US ADI (Acceptable Daily Intake dose) (Bolognesi et al.). Damialis et al. presented automatic, real-time information on concentrations of airborne allergenic pollens that will significantly contribute to the future implementation of accurate, timely, personalized management of allergies. Gosh et al. reported that the results obtained from different studies demonstrate that several metabolic and gene regulation pathways are altered by occupational and environmental exposure to noxious compounds. Another study (Duca el al.) evaluated worker exposure to ultrafine particles and solvents in air, focusing on dermal exposure and biomonitoring. In a pilot study performed in Suriname, Scheepers at al. demonstrated the feasibility of introducing a human biological monitoring program to assess mercury exposure of gold miners and local residents, including children, to reduce the uptake of mercury from the environment. Other projects analyzing diesel exhaust and wood-smoke particles extracts showed that different constituents may contribute to the biological effects of combustion particles (Øvrevik et al.) and that particle composition varies strongly with combustion conditions, which has important implications on population health effects (Pagels et al.). The study by Wierzbicka et al. clearly demonstrated differences in PM1 exposure levels and particle chemical composition between indoor and outdoor environments. Orru et al. showed that near-surface ozone has significant effects on health and that people living in regions with higher levels of benzene, phenol, formaldehyde or PM2.5 had significantly higher odds ($p<0.05$) of experiencing shortness of breath, asthma attacks, long-term cough, phlegm in the lungs, wheezing or chest tightness, as well as heart attacks or angina. Mendes and colleagues plan therefore to assess environmental and health impacts, together with a cost-benefit analysis associated with air pollutant emission reductions, while other partners will focus on environmental tobacco smoke and noise as confounding factors in polluted living and working environments (Teixeira and coworkers). Some project partners plan to evaluate risk communication methods (Macan et al. and Van Damme) or to develop international standards and guidelines relating to new and emerging environmental exposures. In summary, given the evidence of the importance of pollution to the burden of non-communicable diseases, we anticipate a need for regular analyses of long-term human exposures, improvement of biological analysis methods and their performance in comparative analyses, and implementation of this data in future policy assessments.

### References

1. Budnik LT, Adam B, Albin M, Banelli B, Baur X, Belpoggi F et.al. Diagnosis, Monitoring and Prevention of Exposure-related Non-communicable Diseases in living and working environments: DiMoPEx-Project attempts to close the gaps between the impacts of environmental exposures on human health. Submitted  to Journal of Occupational Medicine and Toxicology/ biomedcentral [revised version submitted Dec 20, 2017]
2. European Cooperation on Science and Technology (COST). COST Action DiMoPEx: (Memorandum of understanding) [Internet]  http://www.cost.eu/COST_Actions/ca/CA15129 Accessed December 8, 2017.
3. Scheepers PTJ, Goën T. Diagnosis, Monitoring and Prevention of Exposure-related Non-communicable Diseases – Searching for unique signatures. Pan European Networks: Science & Technology [Internet] 2017 [cited 2017 Dec 8];22:130-3. Available from: http://www.paneuropeannetworks.com/health/searching-for-signatures/
4. 7 million premature deaths annually linked to air pollution [news release]. Geneva: World Health Organization. March 25 2014. Available at: http://www.who.int/mediacentre/news/releases/2014/air-pollution/en/ Accessed December 8, 2017
5. Landrigan PJ, Fuller R, Acosta  NJR, Adeyi O, Arnold R, et al. The Lancet Commission on pollution and health. The Lancet [Internet] 2017 Oct 19 [cited 2017 Dec 1];386:No. 10006 Available from: http://www.thelancet.com/commissions/pollution-and-health Subscription required to view

The following is a list of abstracts presented at the 2th International DiMoPEx (Diagnosis, Monitoring and Prevention of Exposure-related Non-communicable Diseases), all working groups, Conference held on October 30-31, 2017 at Ramazzini Institute, Bentivoglio, near Bologna, Italy.

Abstracts are presented in alphabetical order by corresponding author last name.

Neither the conference organizers or the *Journal of Health and Pollution* can be held responsible for inaccuracies or errors in any included abstracts.

For further information about the conference please visit:

**http://dimopex.eu/wp-content/uploads/2017/09/Flyer_DiMoPEx-Conference_30-31Oct2017-2.pdf**

Plenary lecture

### Impact of pollution on planetary health: emergence of an under appreciated risk factor

Philip J. Landrigan
Dean of Global Health, Icahn School of Medicine at Mount Sinai, New York, USA

Pollution-related disease (PRD) is an increasing, and often neglected, global problem. PRD was responsible for 9 million (M) premature deaths in 2015—three times as many deaths as AIDS, tuberculosis and malaria combined. Air pollution was responsible for 6.4M deaths - 2.8M from household air pollution and 4.2M from ambient air pollution. Water pollution caused 1.75M deaths. Occupational pollutants caused 0.85M deaths. Soil pollution, heavy metals and toxic chemicals caused 0.5M deaths. The nature of pollution is changing. Household air and water pollution are decreasing, and deaths from pneumonia and dysentery are down. However, ambient air, chemical and soil pollution are increasing, and non-communicable diseases (NCDs) caused by these modern forms of pollution are on the rise. Pollution causes 25% of all cardiac deaths, 24% of stroke deaths, 60% of COPD deaths, and 27% of lung cancer deaths worldwide. Pollution is highly inequitable; 92% of PRD occurs in low and middle-income countries (LMICs), and in the hardest hit countries, PRD causes over 30% of all deaths. Pollution and climate change are closely linked, as both arise from the same sources. Climate change worsens the health impacts of pollution, and pollution accelerates climate change. However, both can be controlled by similar solutions. PRD causes great economic losses, including health care costs—1.7% of health care spending in high-income countries and up to 7% in LMICs—and lost productivity from disease and premature death. Pollution and PRD are not the inevitable consequences of development. The hypothesis that countries must go through a phase of pollution as they grow is not substantiated. Proven, cost-effective pollution control strategies are available today to countries at every income level. These solutions are based on law, policy and technology, and the most effective eliminate pollution at the source.

The Lancet Commission on Pollution & Health calls on heads of government, international agencies, and major foundations to make the elimination of pollution a high priority; to set firm targets for PRD reduction; to establish data systems for monitoring pollution and PRD; and to end the externalization of pollution by enforcing the 'polluter pays' principle. A new Global Fund for Pollution and Health or fundamental realignment of an existing international program to explicitly address pollution is urgently needed. Pollution control is a winnable battle.

### Dissemination and implementation of new knowledge within the DiMoPEx COST action

Balázs Ádám (1) Lygia Therese Budnik (2)

Corresponding author:
Balázs Ádám
(adam.balazs@sph.unideb.hu)

(1) University of Debrecen, Faculty of Public Health, Department of Preventive Medicine, Debrecen, Hungary
(2) University Medical Center Hamburg-Eppendorf (UKE), Institute for Occupational and Maritime Medicine (ZfAM), Translational Toxicology and Immunology Unit, Hamburg, Germany

### Background

Environmental exposures constitute a major group of risk factors for human health. There is substantial disease burden related to environmental factors, mainly in the form of morbidity and mortality due to chronic non-communicable diseases including cancer, diabetes, chronic cardiovascular, respiratory, and neurological diseases. The DiMoPEx COST Action aims to foster a joint effort of European scientists to address the issue of adverse health effects from environmental exposures and to suggest effective evaluation and management tools.

### Methods

The facilitation and coordination of information transfer among scientists engaged with health risks due to environmental exposures and the effective wide-scale dissemination and implementation of new knowledge constitute a major activity of DiMoPEx.

Specific meetings are used to formulate plans for cooperation within joint research projects and to prepare for new initiatives. Internal knowledge dissemination is also carried out by the organization of training schools and short-term scientific missions between partnering institutions that provide opportunities for capacity-building, primarily for early career investigators.

### Results

The main output of DiMoPEx is the publication of achievements in collecting and critically assessing information, creating new knowledge, and implementing this knowledge by formulating and testing feasible recommendations for the evaluation and management of health risks due to environmental exposures in various electronic and printed media. Apart from the scientific community, decision-makers of topic-related sectoral policies and industries, as well as the general public, are considered important targets of DiMoPEx knowledge dissemination activities.

### Conclusions

Identifying risk factors in the environment and assessing and managing related health risks are the most effective way to reduce the environment-related disease burden. The DiMoPEx COST Action aims to facilitate cooperation within the scientific community, as well as between scientists and decision-makers to achieve this goal.

### Funding Acknowledgments

Supported by EU-COST project DiMoPEx, CA 15129

### Environmental and DNA repair risk factors for breast cancer: potential use as personalized risk assessment for prevention, intervention and prognosis

Xiaojun Fu (1), Xiaojun Shi (1), Wen-He Huang (2), Guo-Jun Zhang (2), Minodora Dobreanu (3), William Au (1,3)

Corresponding author:
William Au
(au.william@umftgm.ro)

(1) Department of Preventive Medicine, Shantou University Medical College, Shantou, China
(2) Cancer Hospital, Shantou University Medical College, Shantou, China.
(3) University of Medicine and Pharmacy, Tirgu Mures, Romania

### Background

Interactions between environmental and genetic factors are a major cause of cancer development. In addition, DNA repair abnormalities have been shown to be mechanistically involved in the development process. For breast cancer, mutations in the BRCA genes involved in DNA repair activities are responsible for cancer in a small percentage of patients. Therefore, we hypothesized that inherent DNA repair variations (deficiency) are involved in the development of breast cancer and poor prognosis for most breast cancer patients.

### Methods

During the first phase of our investigation, we surveyed 372 patients and 419 matched non-cancer controls in China. The survey showed that high body mass indices, low education level, low fruit intake, sedentary lifestyle, and passive exposure to cigarette smoke were significant risk factors. During the second phase of our study, blood lymphocytes from 60 breast cancer patients and 60 matched non-cancer controls were tested using the challenge comet assay. In the assay, lymphocytes were exposed to 100 cGy X-rays in vitro and these cells were therefore challenged to repair the induced damage. The rates of repair of DNA damage were then quantitatively determined using the comet assay for each donor.

### Results

The repair rates for patients and controls were 52.56±29.17 and 40.22±33.11, respectively, ($p = 0.033$). None of our patients had inherited the BRCA mutations. Among patients, those with lower repair rates were significantly associated with poor prognosis: lymph node metastasis, negative ER receptor and over-expressed Her-2.

### Conclusions

Inherent reduction in DNA repair capacity interacts with environmental risk factors to increase the risk for the development of breast cancer and poor prognosis. In addition, repair deficiency is caused by variations in other DNA repair genes, not BRCA genes. The individualized measurement of DNA repair capacity can potentially be used to provide personalized risk assessments, therapeutic decisions and prognosis predictions.

---

### Creating a "Research in the Field of Regulatory Toxicology" (RESoRT) team: IMROH's capacity building project

Veda Marija Varna (1,2), Željka Babić (1,2), Jelena Kovačić (1)

Corresponding author:
Željka Babić
(zbabic@imi.hr)

(1) Institute for Medical Research and Occupational Health (IMROH), Occupational and Environmental Health Unit, Zagreb, Croatia
(2) Institute for Medical Research and Occupational Health, Poison Control Centre, Zagreb, Croatia

### Background

With better insight into chemicals' toxicokinetics and toxicodynamics, and through improved exposure assessment, toxicological science moves away from the "black box" concept. This is paralleled by similar progress in regulatory toxicology, necessitating continuous research into how we can incorporate scientific advances into the risk assessment process and improve the uncertainty analyses within the process.[1-3]

The highly motivated group of researchers plan to build a multidisciplinary team dedicated to scientific research in the field of regulatory toxicology, particularly computational. The use of *in silico* methodology for regulatory purposes is increasing (e.g. substances' registration under Registration, Evaluation, Authorization and Restriction of Chemicals [REACH]),[4] and there is a need for further research and development of models for higher tier endpoints or complex substances.

### Methods

The team will evaluate the robustness and reliability of existing *in silico* models for the hazard assessment of high tier endpoints (e.g. carcinogenicity) within the framework of the EU harmonized classification of substances, and will work on improving the uncertainty quantification of the predictions. Work along this line has already been initiated on low tier endpoints.[5] Trainings and study visits are planned for early career investigators.

In collaboration with EU experts in the field, further research is planned to focus on the development of improved/new *in silico* models for higher tier endpoints, and/or *in silico* models of aggregate human exposure to arsenic from natural sources, adjusted for the modifying effects of genetic and environmental factors.

### Results

The obtained results will be disseminated in the usual ways. The results and gained knowledge are expected to enable the team to prepare research project proposals at the national level, and to participate in collaborative, international projects in this research area.

### Conclusions

The project is expected to result in the establishment of the RESoRT team, able to scientifically contribute to the field of regulatory and predictive toxicology.

### References

1. Tralau T, Oelgeschläger M, Gürtler R, Heinemeyer G, Herzler M, Höfer T et al. Regulatory toxicology in the twenty-first century: challenges, perspectives and possible solutions. Arch Toxicol [Internet] 2015 Jun;89(6):823-50. doi: 10.1007/s00204-015-1510-0. Epub 2015 Mar 28. Available from: https://link.springer.com/article/10.1007%2Fs00204-015-1510-0 Subscription required to view
2. World Health Organization. WHO Guidance document on evaluating and expressing uncertainty in hazard characterization. Harmonization Project, Document No. 11. Available from: http://www.who.int/ipcs/methods/harmonization/uncertainty_in_hazard_characterization.pdf?ua=1 Published 2014. Accessed December 8, 2017
3. Hartung T, FitzGerald RE, Jennings P, Mirams GR, Peitsch MC, Rostami-Hodjegan A et al. Systems Toxicology: Real World Applications and Opportunities. Chem Res Toxicol [Internet] 2017 Apr 17 [cited 2017 Dec 1];30(4):870-82. doi: 10.1021/acs.chemrestox.7b00003. Epub 2017 Mar 31. Available from: https://www.ncbi.nlm.nih.gov/pmc/articles/PMC5396025/pdf/tx7b00003.pdf
4. European Chemicals Agency (ECHA). ECHA Report, The use of alternatives to testing on animals for the REACH Regulation: Third report under Article 117(3) of the REACH Regulation. Published in 2017. Available from: https://echa.europa.eu/documents/10162/13639/alternatives_test_animals_2017_summary_en.pdf/487e2516-0ad0-90a2-a923-96417ffd6b6b Accessed December 8, 2017
5. Varnai VM. Computational toxicology assessment of skin sensitisation – retrospective application to chemicals with classification harmonised at EU level. Systems Toxicology 2016 Conference. Real World Applications and Opportunities. Les Diablerets, Switzerland. Conference programme and abstracts book [Internet], p. 40, 2016 . Available from: https://systox.ch/2016-Les-Diablerets/

---

### Development of the guideline: immunological methods for diagnosis, monitoring and prevention of exposure-related respiratory allergic diseases: IMPRESS

Xaver Baur (1), Cezmi A Akdis (2), Lygia Therese Budnik (3), Maria Jesus Cruz (4), Axel Fischer (5), Ozlem Goksel (6), Meinir Jones (7), Piero Maestreli (8), Xavier Munoz (9), Benoit Nemery (10), Vivi Schlünssen (11,12), Torben Sigsgaard (12), Paul Siegel (13), Cecilie Svanes (14), Claudia Traidl-Hoffmann (15)

Corresponding author:
Xaver Baur
(baur@eomsociety.org)

(1) European Society for Environmental and Occupational Medicine, Berlin, Berlin, Germany

(2) Swiss Institute of Allergy and Asthma Research, UZH, Global Allergy Forum, Davos, Switzerland
(3) University Medical Center Hamburg-Eppendorf (UKE), Institute for Occupational and Maritime Medicine (ZfAM), Translational Toxicology and Immunology Unit, Hamburg, Germany
(4) Servicio de Neumología, Hospital Universitario Vall d'Hebron, Barcelona, España; CIBER Enfermedades Respiratorias (CibeRes)
(5) CharitćUniversitätsmedizin Berlin, Comprehensive Allergy Center, Berlin, Germany
(6) Translational Pulmonology Research Group (EgeTPAG), Ege University, Faculty of Medicine, Department of Pulmonary Medicine, Division of Asthma, Immunology and Allergy, Izmir, Turkey
(7) Faculty of Medicine, National Heart and Lung Institute, Imperial College London, UK
(8) Department of Cardiologic, Thoracic and Vascular Sciences, University of Padova, Padova, Italy
(9) Servicio de Neumología, Hospital Universitario Vall d'Hebron, Barcelona, España; CIBER Enfermedades Respiratorias (CibeRes); Departamento de Medicina, Universitat Autònoma de Barcelona, Barcelona, España.
(10) Department of Public Health and Primary Care, Centre for Environment and Health, University of Leuven, Leuven, Belgium
(11) National Research Center for the Working Environment, Copenhagen, Denmark
(12) Aarhus University, Department of Public Health, Section for Environment, Occupation & Health, & Danish Ramazzini Centre, Aarhus, Denmark.
(13) National Institute for Occupational Safety and Health (NIOSH)/CDC, Health Effects Laboratory Division, Allergy and Clinical Immunology Branch, Morgantown, WV, USA
(14) Department of Occupational Medicine, Haukeland University Hospital, Bergen, Norway; and Professor, Centre for International Health, Department of Global Public Health and Primary Care. University of Bergen, Bergen, Norway
(15) Christine Kühne Center for Allergy Research and Education (CK-CARE), Augsburg and Davos Technical University Munich, UNIKA, Munich, Germany; & Technical University of Munich and Helmholtz Zentrum München, Germany - German Research Center for Environmental Health, UNIKA-T, Chair and Institute of Environmental Medicine, Augsburg, Germany

### Background

Airborne allergens in living and working environments play an important role in eliciting respiratory allergies, including asthma. These non-communicable diseases have dramatically risen with industrialization and urbanization worldwide.[1,2] Nearly 600 asthma-causing occupational agents were identified, around two-thirds of which are airway sensitizers.[3] Every year, new additional allergenic agents in worksites are identified.[3] Some of these agents are found later in consumer products (e.g. genetically engineered enzymes) contributing to a long list of new sensitizing agents.

The diagnostic measurement of specific IgE is crucial, because it is the major step in identifying the precise cause of respiratory sensitization. However, a limited number of allergens for specific IgE testing are available on the market, necessitating in-house testing. A further limitation of specific IgE measurement is the lack of validated preparations for many agents. Currently, there are a number of immunological tests available on the market and in use. The results often diverge considerably and the test results are hard to compare.

### Objectives

Our project group recognizes the need to examine laboratory diagnostic methods with respect to clinical diagnosis, and to establish suitable interactive mechanisms. There is a need for international consensus to serve as an important resource for physicians and laboratories.

### Methods

We will prepare a review on currently available immunological specific IgE antibody tests, define technical standards for such measurements and provide recommendations with a focus on diagnosis of occupational and consumer-related asthma, e.g. due to genetically-modified enzymes in products.[4-6] The recommendations will include in-house testing with indicated allergens, since there are no consistent methods to validate and standardize in-house allergy tests. Quantitative evaluation of allergen-specific IgE requires cut-off values using a suitable non-exposed healthy reference population.

### Conclusions

The outcome of this project will have implications for both environmental and occupational allergies. An international guideline is planned.

### References

1. Ring J, Akdis C, Behrendt H, Lauener RP, Schappi G, Akdis M, et al. Davos declaration: allergy as a global problem. Allergy [Internet] 2012 Feb [cited 2017 Dec 1];67(2):141-3. doi: 10.1111/j.1398-9995.2011.02770.x. Available from: http://onlinelibrary.wiley.com/doi/10.1111/j.1398-9995.2011.02770.x/full
2. Weisel CP. Assessing exposure to air toxics relative to asthma. Environ Health Perspect [Internet] 2002 Aug [cited 2017 Dec 1];110(Suppl 4):527-37. Available from: https://www.ncbi.nlm.nih.gov/pmc/articles/PMC1241201/pdf/ehp110s-000527.pdf
3. Baur X. A compendium of causative agents of occupational asthma. J Occup Med Toxicol [Internet] 2013 May 24 [cited 2017 Dec 1];8(1):15. doi: 10.1186/1745-6673-8-15. Available from: https://www.ncbi.nlm.nih.gov/pmc/articles/PMC3665602/pdf/1745-6673-8-15.pdf
4. Rodrigo MJ, Morell F, Helm RM, Swanson M, Greife A, Anto JM, et al. Identification and partial characterization of the soybean-dust allergens involved in the Barcelona asthma epidemic. J Allergy Clin immunol [Internet] 1990 Apr [cited 2017 Dec 1];85(4):778-84. Available from: http://www.jacionline.org/article/0091-6749(90)90198-D/pdf
5. Hilger C, Swiontek K, Hentges F, Donnay C, de Blay F, Pauli G. Occupational inhalant allergy to pork followed by food allergy to pork and chicken: sensitization to hemoglobin and serum albumin. International archives of allergy and immunology [Internet] 2010 [cited 2017 Dec 1];151(2):173-8. doi: 10.1159/000236008. Epub 2009 Sep 15. Available from: https://www.karger.com/Article/Abstract/236008 Subscription required to view
6. Budnik LT, Scheer E, Burge PS, Baur X. Sensitising effects of genetically modified enzymes used in flavour, fragrance, detergence and pharmaceutical production: cross-sectional study. Occupational and environmental medicine [Internet] 2017 Jan [cited 2017 Dec 1];74(1):39-45. doi: 10.1136/oemed-2015-103442. Epub 2016 Sep 21. Available from: http://oem.bmj.com/content/74/1/39.long Subscription required to view

### Mixed dust pneumoconiosis, more common than previously thought

Xaver Baur (1), Soma Sanyal (2), Jerrold L. Abraham (2)

Corresponding author:
Xaver Baur
(baur@eomsociety.org)

(1) European Society for Environmental and Occupational Medicine, Berlin, Germany
(2) Department of Pathology, SUNY Upstate Medical University Syracuse, New York, USA

### Background

Classical pneumoconioses, asbestosis and silicosis have been radiologically defined and differentiated as reticular-nodular patterns predominantly in the lower lung fields versus small rounded opacities mostly in the upper lung zones, respectively. Pathologically, they have been described as interstitial fibrotic versus nodular disease, respectively. However, it has been increasingly recognized that workers suffer from mixed dust pneumoconiosis with overlapping radiological and pathological patterns due to complex, cumulative inhalational exposures at the workplace. We have diagnosed several such cases in the recent years and are presenting one case where detailed pathological and mineralogic investigations could be performed.

### Results

The 59-year-old smoker reported pneumonia 12 years ago and increasing symptoms of COPD since then. Five years ago his symptoms worsened, he became incapacitated and needed oxygen treatment. Computed tomography exhibited an irregular fibrotic lung pattern and fibrotic pleural lesions, as well as a cavity in his right upper lung lobe. He had worked for nearly 30 years for a company that produced various kinds of refractory material using large amounts of bauxite, silicon carbide, and various recycling materials, including aluminum sheets and ceramic fiber products. Histopathology of the two resected lung segments showed focally infarcted granulomas and chronic inflammation. Special stains for organisms were negative. The lung tissue away from the granulomas showed significant dust deposition including dust macules. No silicotic nodules were seen. The dust itself was mixed, fine and coarse, opaque and birefringent, including welding fume/foundry type particles and a few long thin fibers with a golden brown color, some coated to become ferruginous bodies where asbestos bodies could not be excluded. SEM/EDS (scanning electron microscopy/energy dispersive spectroscopy) analysis showed predominantly Si (silica or silicon carbide [SiC]) and Al particles (consistent with aluminum metal and/or oxide), as well as numerous Al silicates, Ti, and occasional Zr, Nb, V, steel, and Si fibers (consistent with SiC).

### Conclusions

Initially, due to the radiological findings which were interpreted as not typical for pneumoconioses and the presence of granulomas lung tissue, necrotizing sarcoid granulomatosis was diagnosed. The detailed examination revealed that it was mixed dust pneumoconiosis. Diagnostics of interstitial lung disorders should always include a detailed occupational history and consider that the various exposures may considerably modify the resulting morphological lung lesions.

### Adverse effects of glyphosate and Roundup administered at human equivalent doses to Sprague-Dawley rats

Simona Panzacchi (1), Daniele Mandrioli (1), Fabiana Manservisi (1), Eva Tibaldi (1), Daria Sgargi (3), Marcella Spinaci (4), Giovanna Galeati (4), Claudia Bolognesi (5), Giovanni Dinelli (2), Rossella Miglio (3), Angela Lenzi (6), Fiorella Belpoggi (1)

Corresponding author:
Fioella Belpoggi
(belpoggif@ramazzini.it)

(1) Ramazzini Institute, Cesare Maltoni Cancer Research Center, Bentivoglio, Bologna, Italy
(2) Department of Agricultural Sciences, University of Bologna, Italy
(3) Department of Statistical Sciences, University of Bologna, Italy
(4) Department of Veterinary Medical Sciences, University of Bologna, Italy
(5) Ospedale Policlinico San Martino, National Cancer Institute, San Martino-IST Environmental Carcinogenesis Unit, IRCCS Genoa, Italy
(6) Unit of Anatomy, Pathological Histology, Sant'Orsola-Malpighi Hospital, Bologna, Italy

### Background

Glyphosate is a broad-spectrum, post emergent, non-selective, systemic herbicide, which effectively kills or suppresses all plant types. It has agricultural and non-agricultural uses throughout the world. It was registered in over 130 countries as of 2010 and is probably the most heavily used herbicide in the world, with annual global production volume estimate at approximately 600,000 tons in 2008 rising to 720,000 tons in 2012. The International Agency for Research on Cancer (IARC) classified glyphosate as a probable carcinogen to humans (Group 2A), but according to the EFSA (European Food Safety Authority) the evidence is not yet sufficient to declare its carcinogenicity with certainty. Given this state of scientific wavering, it is essential to fully understand the potential harmful effects from this substance, including other non-cancer toxicological endpoints.

### Methods

This study examines whether low-dose exposure to glyphosate and/or its commercial formulation Roundup could be related to health effects in Sprague-Dawley rats with exposure from gestation through adulthood. The doses selected are comparable to those currently admitted in humans in the United States in order to mimic real-life exposure and the studied end-points are related to development, reproduction and toxicology.

### Results

We are able to reproduce real-life exposure in a human-equivalent model. Toxic effects are explored in terms of gene expression, hormone alterations, parameters related to fertility and development, growth trends, as well as toxic effects in target organs.

### Conclusions

This pilot study, designed principally to form the basis for a new integrated long term study, provided information and some evidence that maternal exposure to commonly used glyphosate-based herbicides could have harmful effects on different end-points other than cancer.

---

### In vivo micronucleus assay in bone marrow of Sprague-Dawley rats treated with glyphosate and Roundup at human equivalent doses

Claudia Bolognesi (1), Paola Roggieri (1), Andrea Compalati (1), Simona Panzacchi (2), Fabiana Manservisi (2), Daniele Mandrioli (2), Fiorella Belpoggi (2)

Corresponding author:
Claudia Bolognesi
(claudia.bolognesi@hsamartino.it)

(1) Ospedale Policlinico San Martino, San Martino-IST Environmental Carcinogenesis Unit, IRCCS Genoa, Italy
(2) Ramazzini Institute, Cesare Maltoni Cancer Research Centre, Bentivoglio, Bologna, Italy

### Background

Glyphosate is one of the most widely used pesticides globally, with more than 750 glyphosate formulations on the market in 130 countries. The evidence from the literature shows that glyphosate doesn't induce gene mutations, but inconsistent results have been reported with *in vitro* and *in vivo* chromosomal damage assays. A recent systematic review reported the induction of micronuclei (MN) by glyphosate and its formulations with large differences across species, types of cells analyzed and routes of administration. As a part of the pilot study on glyphosate/Roundup toxicity ongoing in Cesare Maltoni Cancer Research Center, the frequency of MN was evaluated in bone marrow of rats treated from embryos at the US Acceptable Daily Intake (ADI).

### Methods

The bone marrow was flushed out from the femurs of rats and dispersed in fetal bovine serum and pooled. Slides were prepared by cell dropping and stained with acridine orange. The MN frequency was determined by analyzing the number of micro-nucleated polychromatic erythrocytes (MN-PCEs) in coded slides from each of 8 rats/sex/group. The PCE:NCE (normochromatic erythrocytes) ratio was calculated to evaluate the cytotoxic effect associated with exposure.

### Results

No statistically significant increase of MN-PCEs was observed in any group of 13-week post weaning treated rats compared with controls. A statistically significant increase ($p< 0.001$) of MN-PCEs was detected in the 6-week post weaning. Roundup-treated animals showed a mean MN-PCE/1000 PCEs: 4.66 ± 1.21 versus 3.25 ± 0.98 in controls. No variation in PCE/NCE ratio was evident in any group.

### Conclusions

The results of our study show a weak increase of MN frequency in the bone marrow of rats treated with Roundup from embryos until 6 weeks after weaning. However the absence of toxicity evaluated as depression of the PCE/NCE ratio does not allow evidence of exposure of the bone marrow.

### Outgassing chemicals found in globally transported products: Characteristics of toxic hazards desorption

Lygia Therese Budnik (1), Sabrina Gadau (1), Lars Andernach (2), Bärbel Brunswig-Spickenheier (1), Harald Jungnickel (2), Andreas Luch (2), Peter Laux (2)

Corresponding author:
Lygia Therese Budnik
(L.Budnik@uke.de)

(1) University Medical Center Hamburg-Eppendorf (UKE), Institute for Occupational and Maritime Medicine (ZfAM), Translational Toxicology and Immunology Unit, Hamburg, Germany
(2) German Federal Institute for Risk Assessment (BfR), Department of Chemical & Product Safety, Berlin, Germany
(3) Research Department Cell and Gene Therapy, Clinic for Stem Cell Transplantation, University Medical Center Hamburg-Eppendorf, Hamburg, Germany
(4) Department of Chemical and Product Safety, German Federal Institute for Risk Assessment (BfR), Berlin, Germany
(5) German Federal Institute for Risk Assessment (BfR), Department of Chemical & Product Safety, Berlin, Germany

### Background

Fumigation of transport containers is a common practice to protect stored products from pests. Products/goods can also be tainted by additional hazardous substances. However, little is known about desorption times of these highly toxic substances found in container air. In our previous study we showed that mean amounts of benzene in container air exceeded 698-fold recommended exposure limits (REL) and the concentration of 1,2-dichloroethane (EDC, ethylene dichloride) residues was 4.5-fold higher than the corresponding REL.[1,2] More than 90% of all containers showed toluene residues higher than its REL.[1,2] To shed light on the outgassing properties and the behavior of fumigants on various products, we treated selected products with phosphine, methyl bromide and

EDC[1,2] and observed that classical fumigants like methyl bromide and phosphine outgassed from packaging materials and textiles in a matter of days, while a desorption time of several months was observed for EDC.[1] Now we present data from the follow up study.

### Methods

Shoes were experimentally fumigated under controlled laboratory conditions with EDC, toluene and dichloromethane (DCM, methyl chloride), n=3 times. Outgassed air samples were analyzed by thermal desorption/2D gas chromatography coupled to mass spectrometry and flame photometric detection (TD-2D-GC-MS/FPD).[1,2] To investigate possible interactions between gases and constituents of the products, non-gas-treated and fumigated samples of wrapping paper were analyzed by ToF-SIMS to examine interactions of the fumigant with molecules of the consumer product to reflect the adhesion of the fumigants to the product surface.[1]

### Results

To reach values below the respective REL 75% percentile, toluene was outgassing for 696 h [max 840 h], EDC was outgassing for 654 h [max 770 h] and shoes tainted with DCM were outgassing for 815 h.

### Conclusions

Desorption times of several months were observed for EDC, DCM and toluene. We assume that within the indicated time period, shoes could well have reached their destination in storage rooms, shops and in private homes of end-consumers, contributing to the mixture of volatiles in indoor air.

### Funding Acknowledgments

Supported by the Strategic Research Program from the German Federal Institute for Risk Assessment, BfR (BfR Project 8124797 to LTB; BfR Project 7031564 to AL).

### References

1. Budnik LT, Austel N, Gadau S, Kloth S, Schubert J, Jungnickel H, et al. Experimental outgassing of toxic chemicals to simulate the characteristics of hazards tainting globally shipped products. PLoS One [Internet] 2017 May 17 [cited 2017 Dec 4];12(5):e0177363. doi: 10.1371/journal.pone.0177363. eCollection 2017. Available from: http://journals.plos.org/plosone/article/file?id=10.1371/journal.pone.0177363&type=printable
2. Austel N, Schubert J, Gadau S, Jungnickel H, Budnik LT, Luch A. Influence of fumigants on sunflower seeds: Characteristics of fumigant desorption and changes in volatile profiles. J Hazard Mater [Internet] 2017 Sep 5 [cited 2017 Dec 4];337:138-47. doi: 10.1016/j.jhazmat.2017.04.070. Epub 2017 Apr 29. Available from: http://www.sciencedirect.com/science/article/pii/S030438941730331X?via%3Dihub Subscription required to view

### DiMoPEx (Diagnosis, Monitoring and Prevention of Exposure-related Non-communicable diseases) project follow up

Lygia Therese Budnik (1), Balazs Adam (2), Maria Albin (3,4), Barbara Banelli (5), Xaver Baur (6), Fiorella Belpoggi (7), Claudia Bolognesi (8), Karin Broberg (4), Per Gustavsson (4), Thomas Göen (9), Axel Fischer (10), Dorota Jarosinska (11++), Fabiana Manservisi (7), Richard O'Kennedy (12), Johan Øvrevik (13), Elizabet Paunovic (11++), Beate Ritz (14), Paul T. J. Scheepers (15), Vivi Schlünssen (16,17), Heidi Schwarzenbach (18), Per E. Schwarze (13), Orla Sheils (19), Torben Sigsgaard (17), Karel VanDamme (20), Ludwine Casteleyn (20)

Corresponding author:
Lygia Therese Budnik
(L.Budnik@uke.de)

(1) University Medical Center Hamburg-Eppendorf, Institute for Occupational and Maritime Medicine (ZfAM), Translational Toxicology and Immunology Unit, Hamburg, Germany
(2) University of Debrecen, Faculty of Public Health, Department of Preventive Medicine, Debrecen, Hungary
(3) University of Lund, Division of Occupational and Environmental Medicine, Lund, Sweden
(4) Karolinska Institutet, Institute of Environmental Medicine (IMM), Stockholm, Sweden.
(5) Ospedale Policlinico San Martino, National Cancer Institute, Tumor Epigenetics Unit, IRCCS and University of Genoa, DISSAL, Genoa, Italy.
(6) European Society for Environmental and Occupational Medicine, Berlin, Germany
(7) Ramazzini Institute, Cesare Maltoni Cancer Research Center, Bentivoglio, Bologna, Italy
(8) Ospedale Policlinico San Martino, National Cancer Institute, San Martino-IST Environmental Carcinogenesis Unit, IRCCS Genoa, Italy
(9) Friedrich-Alexander-University Erlangen-Numberg, Institute and Outpatient Clinic of Occupational, Social and Environmental Medicine, Erlangen, Germany,
(10) Charité Universitäts Medizin, Institute of Occupational Medicine, Berlin, Germany
(11) WHO European Centre for Environment and Health, Bonn, Germany
(12) Dublin City University, Biomedical Diagnostics Institute, Dublin, Ireland
(13) Norwegian Institute of Public Health, Oslo, Norway
(14) University of California Los Angeles (UCLA),Center for Occupational and Environmental Health, Fielding School of Public Health (FSPH), Los Angeles, USA
(15) Radboud University Medical Center, Radboud Institute for Health Sciences, Radboudumc Nijmegen, the Netherlands
(16) National Research Center for the Working Environment, Copenhagen, Denmark
(17) Aarhus University, Department of Public Health, Section for Environment, Occupation & Health, & Danish Ramazzini Centre, Aarhus, Denmark.
(18) Department of Tumor Biology, University Medical Center Hamburg-Eppendorf, Hamburg, Germany
(19) Department of Histopathology, St James's Hospital and Trinity Translational Medicine Institute, Trinity College, Dublin, Ireland
(20) University of Leuven, Center for Human Genetics and Center for Environment and Health, Leuven, Belgium

++Elizabet Paunovic and Dorota Jarosinska are staff members of the World Health Organization (WHO) Regional

Office for Europe. The authors alone are responsible for the views expressed in this publication and they do not necessarily represent the decision or stated policy of the World Health Organization

**Background**

Research addressing the links between environmental exposures and disease prevalence is key for preventing increases in non-communicable disease (NCD; http://dimopex.eu/NCD) morbidity and mortality. However, because of the long latency and chronic course of some diseases and the necessity to address cumulative exposures over very long periods, it is often difficult to identify causal environmental exposures.

**Methods**

The EU-funded project "Diagnosis, Monitoring and Prevention of Exposure Related Non-communicable Diseases" (DiMoPEx) aims to develop new concepts for a better understanding of health-environment (including gene-environment) interactions in the etiology of NCDs.
The overarching idea is to teach early career investigators and to train senior scientists/physicians through interdisciplinary exchange to include efficient and valid exposure assessments in cooperative research projects as well as how to apply this knowledge in public health initiatives.

**Results**

DiMoPEx partners have identified some of the emerging research needs, which include the lack of evidence-based exposure data, the need for suitable animal models reflecting the human life-span and low dose cumulative exposures. Biomarkers of early effect and susceptibility and detection of disease-specific genetic aberrations and epigenetic modulations may elucidate the aberrant regulation of disease-specific signaling pathways. Utilizing an interdisciplinary approach, including seven working groups, DiMoPEx will focus on aspects of air pollution with particulate matter (including dust and fibers), and on exposures to low dose solvents and sensitizing agents. Biomarkers of early exposures and the associated effects as indicators of disease-derived information will be tested and standardized within individual projects.

**Conclusions**

Risks of some NCDs, like pneumoconioses, cancers and allergies are predictable and preventable. Consequently, preventive actions could lead to a reduction of disease morbidity and mortality for many of the NCDs which are of major public concern. DiMoPEx plans to catalyze and stimulate interaction of scientists with policy-makers on exposure-related diseases of concern to society.

**Funding Acknowledgments**

Supported by EU-COST project DiMoPEx, CA 15129

### Life-course exposure and frailty syndrome in older adults

Bruna Lage (1,2), Armanda Teixeira-Gomes (2,3), Susana Silva (1), Filipa Esteves (1,3), Joana Carvalho (4), João Paulo Teixeira (1,3), Solange Costa (1,5)

Corresponding author:
Solange Costa
(solange.costa2@gmail.com)

(1) Environmental Health Department, National Institute of Health, Porto, Portugal
(2) Instituto de Ciências Biomédicas Abel Salazar (ICBAS-UP), Universidade do Porto, Porto, Portugal
(3) EPIUnit - Instituto de Saúde Pública, Universidade do Porto, Porto, Portugal
(4) Research Centre in Physical Activity, Health and Leisure (CIAFEL), Faculty of Sport, Universidade do Porto, Porto, Portugal
(5) Instituto de Ciências, Tecnologias e Agroambiente (ICETA), Universidade do Porto, Porto, Portugal

### Background
The prevention of frailty in old age is one of the key actions identified in the Horizon 2020 Framework Program. Frailty is a multidimensional syndrome characterized by increased vulnerability and functional decline that may be reversed, but if not addressed, can lead to long-term disability and hospitalization. The potential to reduce the prevalence of some major diseases is driving research to understand the totality of exposures over the course of our lifetime. The data presented are preliminary results of the BioFrail project aiming to identify a) new cellular and molecular biomarkers associated with frailty and b) risk factors related to frailty syndrome aetiology, namely environmental and lifestyle factors (e.g. outdoor and indoor air quality).

### Methods
A total of 61 older adults (≥ 65 years old) from Porto (Portugal) were engaged in this study. Frailty status was assessed via Fried's frailty model. Life-time exposure was evaluated by a life-course exposure questionnaire (LTQE). Fireplace use, farming activity, home ventilation and household proximity to traffic were some of the factors analyzed. A job-exposure matrix was also applied to assess the occupational risk to known hazard compounds. DNA damage and oxidative stress endpoints were measured through comet assay in whole blood samples.

### Results
The study population was classified as 47.5% robust, 49.2% pre-frail and 3.3% frail. A significantly higher prevalence of second-hand smokers was found in the pre-frail group compared to the robust group. No significant differences were found in basal DNA damage and oxidative damage between groups. Regarding the influence of exposure-related parameters, a significant effect was obtained for living near farming operations (within the robust group) and living near industrial areas (within the pre-frail group).

### Conclusions

Although the results had some positive findings, it is important to note that the size of the study population limits the value of the data and restricts possible conclusions, and larger studies are needed to confirm these results.

### Funding Acknowledgements

Solange Costa and Armanda Gomes are supported by the Portuguese Foundation for Science and Technology (FCT) under the grants SFRH/BPD/100948/2014 and SFRH/BD/121802/2016 respectively. The authors also acknowledge the contribution of the Project NORTE-01-0145-FEDER-000010 – Health, Comfort and Energy in the Built Environment (HEBE), co-financed by Programa Operacional Regional do Norte (NORTE2020), through Fundo Europeu de Desenvolvimento Regional (FEDER).

---

### Determining actual exposures to allergenic pollen and the need for automatic, real-time pollen monitoring

Athanasios Damialis (1,2), Franziska Häring (1), Manuel Glaser (3), Jens O. Brunner (3), Claudia Traidl-Hoffmann (1,2)

Corresponding author:
Athanasios Damialis
(thanos.damialis@tum.de)

(1) Technical University of Munich and Helmholtz Zentrum München, Germany - German Research Center for Environmental Health, UNIKA-T, Chair and Institute of Environmental Medicine, Augsburg, Germany
(2) Christine Kühne Center for Allergy Research and Education (CK-CARE), Augsburg and Davos Technical University Munich, UNIKA, Munich, Germany
(3) University of Augsburg, Faculty of Business and Economics, UNIKA-T, Chair of Health Care Operations/Health Information Management, Augsburg, Germany

### Background

To date, high-risk pollen exposure alerts have been provided only via pollen season forecasting models and conventional monitoring methods that are laborious and not time-effective. The aim of this study was to compare and evaluate airborne pollen exposure using a novel automatic, real-time pollen sampler vs. conventional sampler. The questions to be answered are: what is the genuine exposure to allergenic pollen and when?

### Methods

Airborne pollen has been monitored in Augsburg, Germany since 2015, using a novel automatic Bio-Aerosol Analyzer together with a network of conventional Hirst-type volumetric traps. Both techniques provided measurements on an hourly scale. The yearly abundance of all recorded pollen types was estimated, as well as the start, peak and end of each pollen season. Moreover, circadian pollen abundance patterns were assessed so as to obtain operational alerts on high-risk hours within each day. Comparisons between the two sampling methods aimed to determine the most accurate technique of assessing genuine pollen exposure.

### Results

It was found that different sampling means detected similar diversity, but variable amounts of pollen yearly. In many cases, the start, peak and end of the pollen season were satisfactorily predicted, especially for the allergenic *Betula* (birch) and *Poaceae* (grasses). The automatic pollen sampler usually estimated higher amounts of pollen in the air. Both sampling techniques were equally capable of identifying the daily pollen peaks in each season. All measurements were broadcasted real-time via the Institute's webpage (www.unika-t.de/pollenflug/).

### Conclusions

Automatic, real-time information on concentrations of airborne allergenic pollen will significantly contribute to the implementation of accurate, timely, personalized management of allergies in the future. However, it is still unclear what the actual exposure to allergenic pollen is, or when it occurs, as this information needs to be integrated with real-life, personalized symptom scores of allergic patients.

---

### Thyroid cancer incidence around Belgian nuclear sites, 2006-2014

Claire Demoury (1), Harlinde De Schutter (2), Michel Sonck (3), Tom De Smedt (1), Nancy Van Damme (2), Lodewijk Van Bladel (3), An Van Nieuwenhuyse (1)

Corresponding author:
Claire Demoury
(claire.demoury@wiv-isp.be)

(1) Scientific Institute of Public Health, Health and Environment unit, Brussels, Belgium
(2) Belgian Cancer Registry, Brussels, Belgium
(3) Federal Agency for Nuclear Control, Brussels, Belgium

### Background

In Belgium, the radiological incident that happened in Fleurus in 2008 raised public concern regarding the possible health effects of residing near nuclear sites. In response, the authorities ordered a nation-wide study. Some nuclear installations are a potential source of emissions of radioiodines and thyroid cancer is known to occur after exposure to these substances. We investigated the incidence of thyroid cancer around Class I Belgian nuclear sites (Tihange, Fleurus, Mol-Dessel and Doel).

### Methods

An ecological study was performed at the level of the statistical sectors, the smallest defined basic area unit. 7,285 incident cases of thyroid cancer registered from 2006 to 2014 were included. The distance between the center of the statistical sector and each of the four Belgian nuclear sites was calculated to define the proximity area. Rate ratios of thyroid cancer were estimated using Poisson regression.

### Results

The Poisson regression was adjusted for age, sex and region. Interactions between sex and age and between region and year were included in the model to deal with differences in the incidence of thyroid cancer. Rate ratios of thyroid cancer were higher in the vicinity of the Fleurus nuclear site; 1.29 (95%CI: 1.06; 1.57), 1.17 (95%CI: 1.04; 1.31), 1.14 (95%CI: 1.03;1.25) and 1.08 (95%CI: 0.99;1.18) at 5 km, 10 km, 15 km and 20 km around the nuclear site, respectively. No higher rates were observed in the proximity of other nuclear sites.

### Conclusions

Rate ratios of thyroid cancer were higher in the vicinity of the Fleurus nuclear installation, a major production site of radioiodines. Analyses based on the modeling of the radioactive discharges due to the nuclear sites are ongoing. If these results are confirmed, this study illustrates the need for further studies examining this increased incidence.

---

### Air pollution generated via unsanitary municipal solid waste landfill in the vicinity of Novi Sad, Vojvodina

Maja Djogo, Jelena Radoni, Mirjana Vojinović[1] Miloradov, Maja Sremački[1], Ivana Mihajlović,[1] Dejan Ubavin

Corresponding author:
Maja Djogo
(majadjogo@uns.ac.rs)

(1) University of Novi Sad, Technical Science faculty, Department of Environmental Engineering and Occupational Safety and Health, Novi Sad, Serbia

### Background

In Serbia, average air contamination is two times higher than recommended and three times higher than in developed European countries. The data show that globally, for every 100,000 population there are 47 fatalities per year, while in Serbia there are 34. The atmosphere is a very fluid medium. Every natural or accidental evaporation, explosion, inputting, combustion and forming of suspended particles that sorbs free gas molecules of pollutants represent sources of contamination. Landfills are generators and reactors of different hazardous and volatile and semi-volatile organic substances such as polycyclic aromatic hydrocarbons (PAHs), polyclirinated byfenils (PCBs), organochlorine pesticides (OCPs), xyloxans and other toxic substances.

### Methods

In order to provide the preliminary information on health risk assessment, cumulative health risk is calculated as a sum of the risk for every individual substance that was included in two-year monitoring programme. Limit values for human exposure to these substances is 1E-6 according to EPA. Exposure where the risk factor is higher than 1E-6 is considered to be significant.

### Results

On a non-sanitary landfill in a suburban part of Novi Sad, persistent, toxic and bio-cumulative hazardous organic gas molecules such PAHs, PCBs, OCPs were detected and health and environmental risk assessments were developed. Two boundary scenarios were designed. In the assessment of the first scenario (real case scenario), used airflow through PUF was 150m$^3$/28d. A lower airflow value of 100m$^3$/28d was used for the second scenario (worst case scenario) prediction.

### Conclusions

Calculated values of total risks for all examined substances in the ambient air of a landfill in Novi Sad are lower than limiting EPA values (1E-6) and do not pose a significant risk to the human population, who live and work near landfill site in Novi Sad, because only the gas phase of ambient air has been analyzed, and various gas pollutant molecules show high sorption potential onto the suspended particles. The aim of the research group from Novi Sad was to examine the processes of mitigation and reduction of ambient air contamination and management of deposited waste mix at a suburban landfill location.

---

### Evaluation of worker exposure to ultrafine particles and solvents: air, dermal and biomonitoring

Radu-Corneliu Duca (1), Horatiu Moldovan (2), Septimiu Voidazan (2), Doina Giurgiu (3), Stelian-Ioan Morariu (4), Matteo Creta (1), Jeroen Vanoirbeek (1), and Lode Godderis (1,5)

Corresponding author:
Radu-Corneliu Duca
(radu.duca@kuleuven.be)

(1) Centre for Environment and Health, Department of Public Health and Primary Care, Leuven, Belgium
(2) University of Medicine and Pharmacy of Tirgu-Mures, Romania
(3) Lucian BlagaćUniversity of Medicine, Sibiu, Romania
(4) VasileGoldisćWest University Arad, Occupational Health Department of the Arad County Clinical Emergency Hospital, Arad, Romania
(5) IDEWE - External Service for Prevention and Protection at Work, Heverlee, Belgium

### Background

Workers are exposed to a range of ultrafine particles (UFP) comprising manufactured nanoparticles and particles coming from natural, human or industrial sources. Furthermore, in most ‘nano-exposed’ workplaces, exposure is not only limited to UFP’s, but also comprises a complex mixtures of chemicals, such as solvents. In this context, simultaneous exposure of workers to UFP and solvents was evaluated within a Romanian factory, within a collaborative project between Belgium and Romania.

### Methods

A cross-sectional study of factory workers, office workers (same factory) and a control group (civil servants in a public institution) was performed. UFP levels in the workplace were evaluated using stationary measurements. Inhalation exposure (using organic vapor monitors), dermal exposure (using activated charcoal cloth patches), and systemic exposure (using urine samples) to solvents were assessed. Volunteers were examined according to the following schedule: physical examination, occupational history based on a specific questionnaire, smoking and alcohol consumption habits. Blood samples were collected to evaluate several inflammatory, oxidative stress and epigenetic biomarkers.

### Results

This collaboration was developed within the DiMoPEx - COST Action project. Currently, the results have been analyzed and will be the subject of several scientific publications. Briefly, respiratory exposure levels differed between the groups for UFP and solvents exposure. Among the factory workers, dermal exposure to solvents was shown to have a direct impact on the total body burden, as reflected by urine biomonitoring. Furthermore, a high simultaneous exposure to both UFP and solvents triggered inflammatory responses in workers.

### Conclusions

This study highlights the complexity of evaluating heterogeneous exposures and determining the causative effects on workers' health.

### Funding

- C3 research project no. 3M160685, financed by KU Leuven, Belgium.
- Research grant no. 235/06.01.2016, financed by the Mediconsult Association, Tîrgu Mureş, Romania.
- Mobility projects for young researchers from the diaspora, no. PN-III-P1-1.1-MCT-2016-0058, financed by UEFISCDI, Romania.

### Impact of environmental and occupational factors on the epigenome

Manosij Ghosh (1), Sara Pauwels (1), Deniz Oner (1), Jelena Bakusic (1), Radu-Corneliu Duca (1), Peter Hoet (1), Lode Godderis (1,2)

Corresponding author:
Manosij Ghosh
(manosij.ghosh@kuleuven.be, lode.godderis@kuleuven.be)

(1) Centre for Environment and Health, Department of Public Health and Primary Care, Leuven, Belgium
(2) IDEWE - External Service for Prevention and Protection at Work, Heverlee, Belgium

### Background

A growing body of evidence demonstrates that genetic and epigenetic factors condition biological responses to occupational and environmental hazards or serve as their targets. These changes remodel an individual's epigenetic terrain and disease susceptibility. These epigenetic changes can be biomarkers of susceptibility, exposure, or effect, depending on how they are used.

### Methods

In this context, our group is involved in identifying epigenetic markers of exposure and effect of several anthropogenic chemicals in an occupational setting. We have studied epigenetic changes induced by particulate matter, prenatal exposure to mercury[1] and solvents in different cell types, as well as in an exposed population. The group has also extensively studied the epigenetic alterations induced by different nanomaterials ($TiO_2$, gold and carbon nanotubes).[2,3] In addition to these anthropogenic compounds, we have studied the influence of nutritional factors on the epigenome of mother and child (MANOE study).[4,5] We are also studying epigenetic changes in the burnout syndrome and chronic pain.

### Results

The results obtained from the different studies illustrate that several metabolic and gene regulation pathways are altered by the occupational and environmental exposure to noxious compounds.

### Conclusions

Furthermore, these results may give critical insights into the epigenetic carcinogenicity of different environmental factors.

### References

1. Cardenas A, Rifas-Shiman SL, Godderis L, Duca R-C, Navas-Acien A, Litonjua AA, et al. Prenatal Exposure to Mercury: Associations with Global DNA Methylation and Hydroxymethylation in Cord Blood and in Childhood. Environ Health Persp [Internet] 2017 Aug 29 [cited 2017 Dec 4];125(8):087022. doi: 10.1289/EHP1467. Available from: https://ehp.niehs.nih.gov/wp-content/uploads/2017/08/EHP1467.alt_.pdf
2. Ghosh M, Öner D, Duca R-C, Cokic SM, Seys S, Kerkhofs S, et al. Cyto-genotoxic and DNA methylation changes induced by different crystal phases of TiO2-np in bronchial epithelial (16-HBE) cells. Mutat Res [Internet] 2017 Feb [cited 2017 Dec 4];796:1-12. doi: 10.1016/j.mrfmmm.2017.01.003. Epub 2017 Feb 10. Available from: http://www.sciencedirect.com/science/article/pii/S0027510716301014?via%3Dihub Subscription required to view

3. Öner D, Moisse M, Ghosh M, Duca RC, Poels K, Luyts K, et al. Epigenetic effects of carbon nanotubes in human monocytic cells. Mutagenesis [Internet] 2017 Jan [cited 2017 Dec 4];32(1):181-91. doi: 10.1093/mutage/gew053. Epub 2016 Nov 13. Available from: https://academic.oup.com/mutage/article-abstract/32/1/181/3865763?redirectedFrom=fulltext Subscription required to view
4. Pauwels S, Ghosh M, Duca RC, Bekaert B, Freson K, Huybrechts I, et al. Maternal intake of methyl-group donors affects DNA methylation of metabolic genes in infants. Clin Epigenetics [Internet] 2017 Feb 17 [cited 2017 Dec 4];9:1-16. doi: 10.1186/s13148-017-0321-y. eCollection 2017. Available from: https://www.ncbi.nlm.nih.gov/pmc/articles/PMC5297118/pdf/13148_2017_Article_321.pdf
5. Pauwels S, Ghosh M, Duca RC, Bekaert B, Freson K, Huybrechts I, et al. Dietary and supplemental maternal methyl-group donor intake and cord blood DNA methylation. Epigenetics [Internet] 2017 Jan 2 [cited 2017 Dec 4];12(1):1-10. doi: 10.1080/15592294.2016.1257450. Epub 2016 Nov 10. Available from: https://www.ncbi.nlm.nih.gov/pmc/articles/PMC5270634/pdf/kepi-12-01-1257450.pdf

### Funding
FWO, European Respiratory Society, Stichting tegen kanker-Belgi

---

### Relationships between respiratory allergy symptoms and natural pollen exposure

Stefanie Gilles (1,4), Mehmet Gökkaya (1), Sebastian Bezold (1,2), Franziska Häring (1), Nikolaos Bounas-Pyrros (2), Isabelle Beck (1), Gertrud Hammel (1), Marianne Thomas (1), Adam Chaker (3), Avidan Neumann (1), Athanasios Damialis (1), Claudia Traidl-Hoffmann (1,4)

Corresponding author:
Stefanie Gilles
(stefanie.gilles@tum.de)

(1) Technical University of Munich and Helmholtz Zentrum München, Germany - German Research Center for Environmental Health, UNIKA-T, Chair and Institute of Environmental Medicine, Augsburg, Germany
(2) Technical University of Munich, Klinikum Rechts der Isar, Department of Dermatology and Allergy, Munich, Germany
(3) Technical University of Munich, Klinikum Rechts der Isar, Department of Otolaryngology, Munich, Germany
(4) Christine Kühne Center for Allergy Research and Education (CK-CARE), Augsburg and Davos Technical University Munich, UNIKA, Munich, Germany

### Background
Pollen of birch and grasses are a main cause of respiratory allergies across Europe. However, it remains unclear how allergic immune responses change during natural exposure. Moreover, little is known about the effects of pollen exposure on respiratory symptoms of non-allergic people. The aim of the study was to investigate the relationships between airborne pollen concentrations and symptoms, local and systemic inflammatory mediator production and immunoglobulins, in allergic and non-allergic people.

### Methods
Respiratory symptoms of pollen-allergic and non-allergic human volunteers were monitored daily during 2015-2016 and were regressed against concentrations of airborne pollen. Chemokines and immunoglobulins were measured in nasal secretions and serum at 3-week intervals throughout the year. Daily airborne pollen measurements were made using a volumetric Hirst-type sampler. Multivariate and dynamic regression modelling and time-series analysis were performed to check for relationships between symptoms and airborne pollen concentrations.

### Results

In the non-allergic cohort, in-season nasal immunoglobulin levels were not significantly changed compared to off-season levels. In the allergic rhinitis cohort, in contrast, nasal IgE levels were increased during the pollen season, whereas levels of IgGs and IgA were decreased. Our key finding is that symptoms always correlate positively with higher concentrations of airborne pollen, both for allergic and non-allergic individuals; this relationship is strongest for the previous day's pollen atmospheric load ($p<0.001$, $r=0.61$). Nasal, ocular and pulmonary symptoms were all significantly correlated with pollen exposure in allergic individuals, whereas in non-allergic individuals, only correlations with nasal and ocular symptoms were observed. The observed relationships seemed to be persistent up to almost two weeks after the exposure.

### Conclusions

Pollen exposure modulates the local production of immunoglobulins in pollen-allergic individuals. In contrast, the symptom measurements imply that pollen exposure cause measurable adverse health effects even in non-sensitized people, highlighting the need for real-life, real-time health information services.

---

### Human biomonitoring tools for exposure assessment of organic and inorganic contaminants

Thomas Göen, Johannes Müller, Hans Drexler

Corresponding author:
Thomas Göen
(thomas.goeen@fau.de)

(1) Friedrich-Alexander-University Erlangen- Numberg, Institute and Outpatient Clinic of Occupational, Social and Environmental Medicine, Erlangen, Germany

### Background

Human biomonitoring (HBM) is the most powerful approach for an accurate assessment of the individual exposure to a chemical. It can be applied if an appropriate biomarker for an ethically and acceptably sampled biological material is defined. HBM parameters have been developed for hundreds of chemicals. For over five decades, the Institute and Outpatient Clinic of Occupational, Social and Environmental Medicine of the University of Erlangen-Nuremberg (IPASUM) has explored appropriate HBM parameters, and developed and established efficient HBM methods and offered these tools for application in medical practice as well as research studies worldwide.

### Methods

The presentation covers the spectrum of HBM parameters which are served by IPASUM as tools for the assessment of chemical exposure. The parameters were divided by their inorganic and organic identity, by the industrial and consumer use of the parent compound, by the analytical procedure and by their assessment in a joint analytical procedure. They are evaluated by their specificity and their sensitivity.

### Results

IPASUM provides biomonitoring parameters for about 30 metals and metalloids using urine, blood, plasma and the erythrocyte fraction by AAS, ICP-MS and ICP-MS coupled with chromatography. Moreover, it provides biomonitoring of about 160 pesticides and biocides, 100 organic solvents, 50 polymer monomers and additives, 40 polychlorinated biphenyls, 25 aromatic amines and nitro-compounds, 20 alkylating agents and many other chemicals. Urine, blood, plasma and erythrocyte fraction are used as well. Analyses are performed by HRGC coupled with mass spectrometry, HPLC coupled with UV absorption, fluorescence and mass spectrometry detection. Most analytical procedures enable the quantification of the HBM parameters in the occupational as well as environmental exposure range.

### Conclusions

IPASUM provides HBM for several hundred contaminants. The spectrum covers HBM parameters for inorganic and organic compounds from all prominent application areas. Most analytical procedures enable HBM for workplace settings and environmental exposure scenarios.

### Human biomonitoring parameters for exposure to non-persistent pesticides

Thomas Göen, Johannes Müller, Heike Denghel

Corresponding author:
Thomas Göen
(thomas.goeen@fau.de)

(1) Friedrich-Alexander-University of Erlangen- Numberg, Institute and Outpatient Clinic of Occupational, Social and Environmental Medicine, Erlangen, Germany

### Background

Exposure to pesticides and biocides is very common at agricultural workplaces as well as in the general population. A valid risk assessment of pesticide exposure in occupational applicators and consumers requires human biomonitoring (HBM). Objectives for a reasonable HBM are the identification of existing parameters for human biomonitoring of pesticide exposure, specificity of parameters and the performance of analytical procedures for this purpose.

### Methods
Publications in toxicology, analytical chemistry, occupational and environmental medicine regarding the metabolism and exposure assessment of carbamates, neonicotinoides, organophosphates, phenoxy carboxylic acids, pyrethroids und triazines and glyphosate were reviewed for suitable HBM parameters, available analytical methods and assessment values.

### Results
After systemic absorption, non-persistent pesticide and biocide agents are rapidly excreted via urine either unchanged or after metabolic transformation. Human biomonitoring based on the renal elimination of the unchanged agent is feasible for glyphosate, neonicotinoides and phenoxy carboxylic acids. In contrast, agent-specific biomonitoring of carbamates, organophosphates, pyrethroids and triazines can only performed by the assessment of specific metabolites in urine. The determination of metabolites, which can be derived from several pesticides of the same type, demonstrates an alternative biomonitoring approach, which can be used for assessment of the cumulative exposure to a group of agents. Only for a few parameters do biological assessment values for the evaluation of HBM results exist.

### Conclusions
Currently, human biomonitoring parameters exist for most synthetic pesticides, which can be used for the risk assessment of exposed individuals. Almost all existing analytical procedures enable the determination of pesticide exposure in the occupational as well as environmental exposure range.

---

### Exposure to indoor air pollutants and their effects on chronic respiratory diseases

Ozlem Goksel (1), Fusun Pelit (2), Nur Toreyin (3), Levent Pelit (2), Suriye Ozgur (4), Burak Ordin (5), Tuncay Goksel (1)

Corresponding author:
Ozlem Goksel
(ozlem.goksel@ege.edu.tr, goksel.ozlem@gmail.com)

(1) Ege University Translational Pulmonology Research Group (EgeTPRG), Ege University, Faculty of Medicine, Department of Pulmonary Medicine, Division of Asthma, Immunology and Allergy, Izmir, Turkey
(2) EgeTPRG.Ege University Faculty of Science, Department of Chemistry. Izmir, Turkey
(3) EgeTPRG. Ege University, Department of Public Health, Division of Work Health and Occupational Diseases, Izmir, Turkey
(4) EgeTPRG. Ege University, Faculty of Medicine, Department of Biostatistics and Medical Informatics. Izmir, Turkey
(5) EgeTPRG. Ege University, Faculty of Science, Department of Mathematics, Izmir, Turkey

### Background

Subjects with chronic respiratory diseases such as chronic obstructive pulmonary disease (COPD) and asthma are especially vulnerable to the detrimental effects of air pollutants. We aim to investigate the possible causality between exposure to various indoor pollutants and emerging chronic airway diseases in Izmir City. We also aim to observe possible reflections of these exposures on the patients' breath VOCs analyses.

### Methods

People living at randomly selected homes within 50 m of the border of one of the large highways in Izmir City were targeted.

Step 1.

Observational cross-sectional study: From sampling area some local addresses have been selected with random sampling technique. Those locations consisted of 58 buildings and 398 apartments with 1271 habitants (Population Directorate of Izmir Municipality, 2016). At least 318 samples were targeted, with a minimum 143 sampling size. The main inclusion criteria included spending at least 8 hours in a day in the same house and being resident in that house for at least 1 year. Indoor environment monitoring for PM2.5, PM4.0, and PM10.0, toxic gases, $CO_2$ and selected common VOCs (ethane, penthane, benzene, styrene, tetrachloroethylene, toluene, m-pxylene, o-xylene etc.) from bedrooms of homes for at least 4 hours per day and repeat measurements every 6 months for 3 years were planned.

Step 2.

Nested case control study: Using European Respiratory Health Survey demographic variables, comorbitidites etc. will be recorded and those with significant questionnaire results indicating chronic airway diseases will refer to pulmonology for certain diagnosis. Among subjects with normal survey results, a well-matched control group will be generated. Both study and control groups will be evaluated with exhaled breath analysis for exhaled VOC profiles. Previously described methodology by our chemistry group will also be used for this study.

### Results

The study is ongoing with home indoor environment air measurements at Step 1 as a spin-off project of DiMoPEx.

### Funding Acknowledgments

Supported by EGE University Scientific Research Commission (15-TIP-074)

### Ethical approval

Ege University Ethical Committee (January 2016)

### References

1. Breathprint of severe allergic asthma with SPME-GC-MS Analysis of exhaled air volatile organic compounds (Ege University Translational Pulmonology Research Group [EgeTPRG]). Levent Pelit, Ozlem Goksel, Tugberk Dizdas, Ilknur Bagatir, Fusun Pelit, Hasan Ertas, Tuncay Goksel, Burak Ordin, Arif Gürsoy, Durmus Ozdemir, Munevver Erdinc, F. Nil Ertas. European Respiratory Journal 2016 48: PA1061; DOI: 10.1183/13993003.congress-2016.PA1061
2. Moleculer characterization of severe allergic asthma patients via exhaled breath volatile organic compounds(Ege University Translational Pulmonology Research Group [EgeTPRG]). Ozlem Goksel, Levent Pelit, Fusun Pelit, Burak Ordin, Arif Gursoy, Durmus Ozdemir, Nil Ertas, Tuncay Goksel. European Respiratory Journal 2017 50: PA4762; DOI: 10.1183/1393003.congress-2017.PA4762
3. Jacquemin B, Siroux V, Sanchez M, Carsin AE, Schikowski T, Adam M, et al. Ambient Air Pollution and Adult Asthma Incidence in Six European Cohorts (ESCAPE). Environ Health Perspect. 2015;123(6):613-21

### Use of administrative databases to investigate the relationships between air quality and risk of non-communicable diseases in Belgium

Ariane Guilbert (1), Koen Simons (1), Lidia Casas Ruiz (2), Bianca Cox (3), Nicolas Bruffaerts (4), Marijke Hendrickx (4), Claire Demoury (1), Dimitrios Profer (1), Mariska Bauwelinck (5), An Van Nieuwenhuyse (1)

Corresponding author:
Ariane Guilbert
(ariane.guilbert@wiv-isp.be)

(1) Scientific Institute of Public Health, Health and Environment Unit, Brussels, Belgium
(2) University of Leuven, Center for Human Genetics and Center for Environment and Health, Leuven, Belgium
(3) Hasselt University, Center for Environmental Sciences, Hasselt, Belgium
(4) Scientific Institute of Public Health, Mycology and Aerobiology Unit, Brussels, Belgium
(5) Interface Demography, Sociology Department, Vrije Universiteit Brussel, Brussels, Belgium.

### Background

Belgian authorities have developed several systems ensuring the near systematic registration of medication sales, hospitalizations or deaths occurring in its territory. Various information is collected by health professionals (date, nature, underlying health causes, socio-demographic characteristics of the individual, etc.) and associated with a unique identifier, specific to each Belgian resident. Simultaneously, monitoring of air quality, climate conditions are ensured by specialized organizations. Initially developed for policy planning, this information is increasingly being used for research purposes.

### Methods

Various studies based on these databases have been conducted at the Belgian Institute of Public Health. These registers have been explored in an isolated way such as to analyze the evolution of asthma medication sales, hospitalization and mortality rates through time. They have also been frequently merged to analyze the relationships 1) between daily concentrations of air pollutants and asthma/COPD medication sales[1]; 2) between daily counts of outdoor aeroallergens and allergy medication sales or asthma hospitalizations[2]; 3) between heat and mortality, etc. Time series are usually used, allowing the adjustment for confounders.

### Results

These approaches highlighted 1) significant associations between $PM_{10}$, $NO_2$ and asthma/COPD medication sales[1]; 2) noteworthy increases in allergy medication sales and asthma hospitalizations in the case of high grass or birch pollen concentrations in Brussels[2]; and 3) significant associations between high daily temperatures and daily mortality in the city of Antwerp. Trends were detailed by age and gender subgroups through stratification.

### Conclusions

Use of national administrative databases is a cost-efficient method to investigate public health issues in Belgium. It allows to work with large population samples and data collected in a standardized and reliable way. New projects are ongoing, including the linkage of mortality data with air pollution levels (European ELAPSE project), retrospective cohort or closer investigation of multimorbidity issues.

### References

1. Casas L, Simons K, Nawrot T, Brasseur O, Declerck P, Buyl R, et al. Respiratory medication sales and urban air pollution in Brussels (2005 to 2011). Environ Int [Internet] 2016 Sep [cited 2017 Dec 4];94:576-82. doi: 10.1016/j.envint.2016.06.019. Epub 2016 Jun 23. Available from: http://www.sciencedirect.com/science/article/pii/S0160412016302379?via%3Dihub Subscription required to view
2. Guilbert A, Simons K, Hoebeke L, Packeu A, Hendrickx M, De Cremer K, et al. Short-Term Effect of Pollen and Spore Exposure on Allergy Morbidity in the Brussels-Capital Region. Ecohealth [Internet] 2016 Jun [cited 2017 Dec 4];13(2):303-15. doi: 10.1007/s10393-016-1124-x. Epub 2016 May 12. Available from: https://www.ncbi.nlm.nih.gov/pmc/articles/PMC4996865/pdf/10393_2016_Article_1124.pdf

---

### Exposure to traffic-related air pollutants and health conditions: pilot study in Brussels, Belgium

Ariane Guilbert (1), Koen De Cremer (1), Billie Heene (2), Priscilla Declerck (2), Olivier Brasseur (2), An Van Nieuwenhuyse (1)

Corresponding author:
Ariane Guilbert
(ariane.guilbert@wiv-isp.be)

(1) Scientific Institute of Public Health, Health and Environment Unit, Brussels, Belgium
(2) Department Laboratory and Air Quality, Brussels Environment, Brussels, Belgium

### Background

Belgium faces levels of air pollution regularly exceeding the WHO guidelines. The situation is especially concerning in the capital where traffic and heating systems represent the main sources of pollutants and few data on their health effects exist for this area. In that context, public authorities set up a pilot study aiming to assess exposure to traffic-related air pollutants and health conditions in a sample of the Brussels population.

### Methods

A cross-sectional approach was adopted and 48 persons working in Brussels parks were enrolled in 2016-2017. Each participant was followed for four days, during which his or her exposure to black carbon particles, polycyclic aromatic hydrocarbons (PAHs) and benzene was measured using aethalometers, urinary biomarkers (1-hydroxypyrene, 1-naphthol, 2-naphthol, S-phenylmercapturic acid [SPMA]) and questionnaires. In addition, data on respiratory, cardiac health and oxidative stress were collected with questionnaires, as well as respiratory and urinary biomarkers (exhaled nitric oxide, 8-hydroxydeoxyguanosine [8-OHdG]). Regression models were then developed to investigate associations between exposure and health parameters.

### Results

Exposure to black carbon stemming from traffic strongly varied according to working site, ranging from 0.20 μg/$m^3$ in parks located on the outskirts of the capital to 2.59 μg/$m^3$ in parks close to high traffic streets. No association with PAHs biomarkers and no detectable SPMA concentration were observed. A total of 35.4% of participants reported various respiratory problems and 4 individuals suffered from respiratory inflammation. 8-OHdG concentrations varied between 7.05 μg/g creatinine and 17.36 μg/g creatinine among non/ex-smokers. Noteworthy linear relationships were shown between exposure to black carbon and respiratory inflammation/oxidative stress.

### Conclusions

Despite some limitations due to the pilot nature of the study (sample characteristics, data collection period, etc.), relevant results are observed. This work paves the way for bigger projects to confirm the detected trends. Other populations could be involved and data collection methods could be optimized using additional electronic systems.

### Neurological deficits after long-term pyrethroid exposure

Martin Rune Hassan Hansen (1), Erik Jørs (2), Flemming Lander (2), Guido Condarco (3), Fróði Debes (4,5), Noemi Tirado Bustillos (6), Vivi Schlünssen (1,7)

Corresponding author:
Martin Rune Hassan Hansen
(martinrunehassanhansen@ph.au.dk)

(1) Aarhus University, Department of Public Health, Section for Environment, Occupation and Health & Danish Ramazzini Centre, Aarhus, Denmark
(2) Odense University Hospital, Department of Occupational Medicine, Odense, Denmark
(3) The Plagbol Foundation, La Paz, Bolivia

(4) University of Southern Denmark, Department of Public Health, Research Unit of Environmental Medicine, Odense, Denmark
(5) The Faroese Hospital System, Department of Occupational and Public Health, Tórshavn, Faroe Islands
(6) Universidad Mayor de San Andrés, Faculty of Medicine, Genetics Institute, Genetic Toxicology Unit, La Paz, Bolivia
(7) National Research Centre for the Working Environment, Copenhagen, Denmark

### Background

Pyrethroid pesticides have been suggested to be a cause of Parkinson disease and other neurodegenerative diseases.

### Methods

A cross-sectional study was conducted among 120 Bolivian public health vector program spray men, primarily exposed to pyrethroids. Pesticide exposure and central nervous system (CNS) symptoms were determined by structured interview, whereas neuromotor and neurocognitive performance were assessed using the computerized Behavioral Assessment and Research System and CATSYS system.

### Results

Individuals exposed to higher levels of pesticides reported significantly more CNS symptoms (adjusted odds ratio per quintile of cumulative exposure = 2.01 [1.22-3.31]). There was no association seen between pyrethroid exposure and neuromotor performance. Higher spraying intensity was associated with significantly worse neurocognitive performance in structural equation models (adjusted β per quintile = −0.405 [−0.660 to −0.150]), and workers only exposed to pyrethroids performed worse than workers also exposed to other pesticides (adjusted β = −1.344 [−2.224 to −0.464]).

### Conclusions

Chronic pyrethroid exposure may cause deterioration in neurocognitive performance, and exposure control is recommended.

### References

1. Hansen MRH, Jørs E, Lander F, Condarco G, Debes F, Bustillos NT, et al. Neurological Deficits After Long-term Pyrethroid Exposure. Environmental Health Insights [Internet] 2017 Mar 29 [cited 2017 Dec 4];11:1-11. doi:10.1177/1178630217700628. Available from: https://www.ncbi.nlm.nih.gov/pmc/articles/PMC5398229/pdf/10.1177_1178630217700628.pdf

### On the need for a standardized human biomonitoring protocol for in-flight incidents ("fume events")

Astrid Rita Regina Heutelbeck (1), Xaver Baur (2), Fiorella Belpoggi (3), Lygia Therese Budnik (4), Jonathan Burdon (5,6), David Gee (6,8), C. Vyvyan Howard (7), Susan Michaelis (8), Joakim Pagels (9), Colin L. Soskolne (10), Andrew Watterson (11)

Corresponding author:
Astrid Rita Regina Heutelbeck
(aheutel@gwdg.de)

DiMoPEx Project in a cooperation with the WG 1 (Thomas Göen), WG 2 (Paul Scheepers), WG 4 (Ludwine Casteleyn)

(1) University Medical Center Göttingen University, Institute for Occupational, Social- and Environmental Medicine, Göttingen, Germany
(2) European Society for Environmental and Occupational Medicine, Berlin, Germany.
(3) Ramazzini Institute, Cesare Maltoni Cancer Research Center, Bentivoglio, Bologna, Italy
(4) University Medical Center Hamburg-Eppendorf, Institute for Occupational and Maritime Medicine (ZfAM), Translational Toxicology and Immunology Unit, Hamburg, Germany
(5) St Vincent's Hospital, Melbourne, Australia
(6) Institute of Environment, Health and Societies - Brunel University London, UK
(7) Centre for Molecular Biosciences, University of Ulster, UK
(8) Occupational and Environmental Health Research Group, University of Stirling/ Michaelis Aviation Consulting, West Sussex, England
(9) Lund University, Ergonomics & Aerosol Technology, Lund, Sweden
(10) University of Alberta, Edmonton, Canada & Health Research Institute, University of Canberra, Australia
(11) Occupational and Environmental Health Research Group, Centre for Public Health and Population Health Research University of Stirling, Scotland, UK

### Background

In-flight incidents, called "fume events", are associated with a range of symptoms among those exposed, mostly among susceptible airline crew members. Despite currently ongoing ambient monitoring studies, there are no systematic investigations using clinical or human biomonitoring data to evaluate the direct impact of possible harmful substances on human health and well-being. With respect to exposures, we examine chemicals and mixtures of engine oils, hydraulic and deicing fluids, kerosene and pyrolysis/combustion products contaminating bleed air in normal or accidental failure conditions.[1,2] Blood and/or urine samples of patients suffering from pulmonary, neurological, or cerebral symptoms show the incorporation of different harmful substances, including organophosphates and various volatile organic compounds.[3]

### Methods

The international project group "Fume Events" aims to develop new frameworks for better understanding health-environment interactions of in-flight incidents.

Current needs include: [1] Evaluation of the relevance, if any, of current international standards and limit values for exposure in the aircraft environment; [2] Development of applicable, tailored human biomonitoring strategies to monitor particular normal or accidental circumstances; [3] Evaluation of the analytical standards required for the analyses; and [4] Development of diagnostic algorithms supporting physicians in charge.

### Results

Methods used focus either on dose monitoring, biochemical monitoring or on biological effect monitoring, and underpin previously used methods to determine internal exposure to organophosphates that induce delayed neuropathy, a neurodegenerative disorder in mostly agricultural settings. The symptoms correspond to those detected health disorders in persons affected in the course of fume events.[1,3,4,5] In addition, other substances' impending human toxic potential are described.

Any correlation between symptoms and the possible internal uptake of toxic substances can be derived only if blood and/or urine samples were taken close to the date of a fume event and compared (in a quantitative and qualitative way) with the reference values/samples from unexposed controls. However, initial experiences have shown quantitatively and qualitatively variable results. Neither international (independent) standards, nor limit values exist to be considered for respective substances, or for this specific environment. No reference values exist for the general public.

### Conclusions

Recommendations for the evaluation of appropriate independent human biomonitoring strategies using standardized methods in certified laboratories (comparable results for respective samples from exposure patients and reference controls) are needed. Both clinical and toxicological perspectives are needed and are being sought.

### References

1. Michaelis S, Burdon J, Howard CV. Aerotoxic syndrome: a new occupational disease?. Public Health Panorama [Internet] 2017 June [cited 2017 Dec 8];3(2);198-211. Available from: http://www.euro.who.int/en/publications/public-health-panorama/journal-issues/volume-3,-issue-2,-june-2017
2. Michaelis, S. Contaminated aircraft cabin air. J Biol Phys [Internet] 2011 Dec [cited 2017 Dec 4];11(4):132-45. doi: 10.4024/24MI11A.jbpc.11.04. Available from: http://www.itcoba.net/24MI11A.pdf
3. Heutelbeck A, Bornemann C, Lange M, Seeckts A, Muller MM. Acetylcholinesterase and neuropathy target esterase activities in 11 cases of symptomatic flight crew members after fume events. J Toxicol Env Health, Part A [Internet] 2016 [cited 2017 Dec 4];79(22-23):1050-6. doi: 10.1080/15287394.2016.1219561. Available from: http://www.tandfonline.com/doi/full/10.1080/15287394.2016.1219561 Subscription required to view
4. Pinkerton LE, Hein MJ, Grajewski B, Kamel F, et al. Mortality from neurodegenerative diseases in a cohort of US flight attendants. Am J Ind Med [Internet] 2016 Jul [cited 2017 Dec 4];59(7):532-7. doi: 10.1002/ajim.22608. Epub 2016 May 17. Available from: https://www.ncbi.nlm.nih.gov/pmc/articles/PMC4915549/pdf/nihms-795094.pdf

5. Burdon J. Respiratory symptoms and lung injury after inhaling fumes on aircraft: toxic fumes or hyperventilation? J Biol Phys [Internet] 2014 [cited 2017 Dec 4];14:103-6. doi: 10.4024/21BU14A.jbpc.14.04. Available from: https://www.researchgate.net/publication/277887007_Respiratory_symptoms_and_lung_injury_after_inhaling_fumes_on_aircraft_toxic_fumes_or_hyperventilation Subscription required to view

### Human metabolism and biomonitoring parameters of the plasticizer tri-(2-ethylhexyl) trimellitate

Christine Höllerer, Elisabeth Eckert, Thomas Göen

Corresponding author:
Christine Höllerer
(Christine.hoellerer@fau.de)

(1) Friedrich-Alexander-University of Erlangen- Numberg, Institute and Outpatient Clinic of Occupational, Social and Environmental Medicine, Erlangen, Germany

### Background

As the plasticizer tri-(2-ethylhexyl) trimellitate (TOTM) is increasingly used for soft-PVC medical devices, the aim of our project is to investigate the human metabolism of TOTM and to develop a biomonitoring method for blood and urine.

### Methods

Firstly, the in vitro metabolism of TOTM and the three isomers of di-2-(ethylhexyl) trimellitate (DEHTM), respectively, were investigated using porcine liver esterase. The generation of DEHTM and mono-2-(ethylhexyl) trimellitate (MEHTM) isomers by enzymatic ester hydrolysis was assessed by a new developed LC-MS/MS procedure.[1] Secondly, a dose of 100 mg of TOTM was administered orally to a volunteer. Several blood samples were collected until 48 h after ingestion and the total volume of each urine void was collected until 70 h after ingestion. Both, blood1 and urine samples were processed and analyzed for expected TOTM metabolites by liquid chromatography coupled with ESI-tandem mass spectrometry.

### Results

In the in vitro experiment, the preferred ester hydrolysis products were 2-MEHTM and 1-MEHTM, indicating a regioselective formation of primary degradation products of TOTM. In the in vivo study, TOTM as well as the monoester isomers 1-MEHTM and 2-MEHTM and the diester isomers 1,2–DEHTM and 2,4–DEHTM, respectively, with maximum levels at 5 h, 5 h and 3 h after TOTM intake, were detected in blood. For urine, the monoesters 2-MEHTM and 1-MEHTM as well as four secondary main oxidization products (1-M(5cxEP)TM (1-mono-(2-ethyl-5-carboxypentyl) trimellitate), 2-M(5Cx-EP)TM, 2-M(5hydroxyEH)TM (2-mono-(2-ethyl-5-hydroxyhexy) trimellitate) and 1-M(5hydroxyEH)TM) were detected with maximum levels at 5 h, respectively, after the TOTM intake.

### Conclusions

The results demonstrate a coincident TOTM metabolism pathway under in vitro and in vivo conditions. In fact, TOTM was resorbed and metabolized in the human body, as TOTM and its postulated metabolites were detectable in blood and urine, now enabling the establishment of a biomonitoring procedure for TOTM for the first time.

### References

1. Höllerer C, Müller J, Göen T, Eckert E. Isomeric separation and quantitation of di-(2-ethylhexyl) trimellitates and mono-(2-ethylhexyl) trimellitates in blood by LC–MS/MS. J Chromatogr Sci [Internet] 2017 Sep 1 [cited 2017 Dec 4];1061-1062:153-62. doi: 10.1016/j.jchromb.2017.07.014. Epub 2017 Jul 10. Available from: http://www.sciencedirect.com/science/article/pii/S1570023217304269?via%3Dihub Subscription required to view

### Biological monitoring of workers exposed to carcinogens using the buccal or nasal micronucleus approach

Nancy B Hopf (1), Claudia Bolognesi (2), Brigitta Danuser (1), Pascal Wild (1,3)

Corresponding author:
Nancy B Hopf
(Nancy.Hopf@hospvd.ch)

(1) IST, Institute for Work and Health, Universities of Lausanne and Geneva, Lausanne-Epalinges, Switzerland. Nancy.Hopf@hospvd.ch; Brigitta.Danuser@hospvd.ch
(2) Ospedale Policlinico San Martino, National Cancer Institute, San Martino-IST Environmental Carcinogenesis Unit, IRCCS Genoa, Italy claudia.bolognesi@hsanmartino.it
(3) INRS, French Institute for Research and Safety, Vandoeuvre, France, pascal.wild@inrs.fr

### Background

A biomarker-based approach using micronuclei (MNs) (extranuclear DNA-containing bodies) frequencies in nasal and buccal cells has been proposed to monitor workers exposed to aero-digestive carcinogens to reduce the occurrence of occupational cancer. To assess this non-invasive MN approach, we sought to understand: [1] What are the MN frequency ratios of occupationally exposed over non-exposed populations across studies published in the scientific literature for both buccal and nasal cells; and [2] Which type of exposures give the highest mean MN ratio across studies published in the scientific literature.

### Methods

A systematic literature review was performed with the following search terms: “micronucleus” and/or “micronuclei” in combination with “occupational”, “buccal” and/or “nasal”. The search was last conducted on October 3rd 2017. The MN frequency ratios for buccal and nasal cells were calculated for each study.

### Results

DiMoPEx made this project collaboration possible by bringing together toxicologists, medical doctors, statisticians, and exposure assessors. This multidiscipline approach is needed especially when using biomarkers in population surveys. Our search identified 519 articles that we examined closely. From the 102 studies selected, 128 exposures were identified, which allowed us to compute risk ratios (RRs). These exposures were classified in 12 different exposure groups, which represented either carcinogen type or occupation. The highest summary RRs were obtained for inorganic dusts (4 studies in coal mines or quarries) and formaldehyde (5 exposure groups of hospital lab workers). The large variability across studies could be related to the currently unknown genotoxic agents' mechanisms to alter DNA structure and affect nuclear integrity. Automating the MN cell counting process would also reduce the variability introduced by different laboratories.

### Conclusions

The MN frequency biomonitoring approach is a promising tool, especially among populations exposed to multiple carcinogens simultaneously. This MN frequency tool would be even more valuable if the MN frequencies in buccal and nasal cell methods were standardized and shown to be predictive of cancer risk.

### Development of biomarkers for identification of frailty in the elderly

Vanessa Valdiglesias (1), María Sánchez-Flores (1), Diego Marcos-Pérez (1), Ana Maseda (2), José C. Millán-Calenti (2), Laura Lorenzo-López (2), Eduardo Pásaro (1), Blanca Laffon (1)

Corresponding author:
Blanca Laffon
(blaffon@udc.es)

(1) Universidade da Coruña, DICOMOSA Group, Department of Psychology, Area of Psychobiology, A Coruña, Spain
(2) Universidade da Coruña, Gerontology Research Group, Instituto de Investigación Biomédica de A Coruña (INIBIC), Complexo Hospitalario Universitario de A Coruña (CHUAC), SERGAS, A Coruña, Spain

### Background

Currently, progressive population aging has brought about an increase in age-dependent pathologies and, therefore, a higher possibility of developing disability and/or dependence, along with corresponding socioeconomic and healthcare implications. "Frailty" is a multidimensional syndrome with mental and physical connotations involving an increase in vulnerability and a risk factor for poor health outcomes. Epidemiologic research has led to the identification of a number of risk factors for frailty, including pathologic events closely related to exposure to environmental factors.
Frailty assessment is currently based on phenotypic features, namely unintentional weight loss, exhaustion, low physical activity, slow walking speed and low grip strength.[1] However, a better knowledge of the biological basis of frailty would lead to the development of biological markers which would eventually allow a more objective and earlier identification of frail individuals.

### Methods

Cellular and molecular biomarkers of frailty may include indicators of genomic instability, endocrine and immunological status and oxidative stress status.

### Results

Determining and validating these biomarkers would provide a significant advance in healthcare of older adults since it would facilitate anticipation of frailty states and detection of vulnerable patients prior to clinical manifestations. Together with improvement of quality of life in this population, it would also support personalized healthcare, introduce suitable dependency programs, and reduce socioeconomic and healthcare costs.

### Conclusions

Advances in potentially modifiable risk factors for frailty (including environmental factors) form the basis for translational research efforts aimed at prevention and treatment of frailty in elder adults. Anticipation of the frailty state through the use of cellular and molecular biomarkers provides a powerful tool for implementing effective preventive actions by removing/modifying risk factors.

### Acknowledgments

Funded by Xunta de Galicia (grant numbers ED431B 2016/013, ED431C 2017/49, ED481B 2016/190-0, and FrailNet network IN607C 2016/08).

### References

1. Fried LP, Tangen CM, Walston J, Newman AB, Hirsch C, Gottdiener J, Seeman T, Tracy R, Kop WJ, Burke G, McBurnie MA; Cardiovascular Health Study Collaborative Research Group. Frailty in older adults: evidence for a phenotype. J Gerontol A Biol Sci Med Sci. [internet] 2001 [cited 2017 Dec 4];56:M146-156. Available from: https://academic.oup.com/biomedgerontology/article/56/3/M146/545770

---

### Phosphine exposure in maritime foodstuff transportation: recent logistic chain case study

David Lucas (1), Brice Lodde (2), Solenn Guibourg (2), Gilles Mauguen (3), Dominique Jegaden (1), Christophe Paris (4), Jean-Dominique Dewitte (2)

Corresponding author:
David Lucas
(d.lucas@metrabrest.com)

(1) Occupational Health Service of Iroise, Brest, France
(2) Center of Occupational Health and Environmental Diseases, Brest Teaching Hospital, Brest, France
(3) Regional Institute for Occupational Health Prevention, Rennes, France
(4) University of Rennes, Center of Occupational Health, Rennes, France

### Background

The multiplicity and high frequency of intercontinental maritime routes increases the risk of pest infestations. The Food and Agriculture Organization (FAO) recommends treating goods with fumigants. For foodstuffs, phosphine is frequently used.

### Methods

We described a case of phosphine occupational exposure during corn unloading in a French port on December 2016. Atmospheric measurements were done onboard the vessel, in port silos and trucks. Workers were interviewed in person.

### Results

No docker workers described symptoms of acute exposure, but 4 port silos workers described headaches, nausea and abdominal pain with diarrhea.
The atmospheric level of phosphine in 3 tanks on board the vessel was higher than 20 ppm on the first day of unloading. Levels were recorded at 3.5 and 2.5 ppm in workshops and 1 ppm in a silo. Despite ventilation, levels in two tanks on the vessel were greater than 20 ppm for two days and at 0.1 after four days of active ventilation. At the port facilities, 0.8 ppm of phosphine was still noted in the atmosphere of one silo 13 days later. Surprisingly, we found phosphine in the tipper's trucks, with some levels at 1 ppm.

### Conclusions

With this case, we underline the risk of phosphine exposure in maritime foodstuffs transportation and in port and logistic facilities. For phosphine foodstuffs fumigation, two methods are most frequently used. One method involves powder of aluminum phosphide in long plastic tubes placed on the top of the freight. In the other method, powder in the form of small tablets is introduced during loading of the cargo. When unloading the cargo, tablets and aluminum phosphide come into contact with moisture, initiating a chemical reaction for phosphine emissions. That's why we found high levels at different steps of the logistic chain. In this port, phosphine foodstuffs fumigation by tablets is currently prohibited and measurements of the tank atmosphere are now performed prior to unloading.

---

### Volatile organic compounds in containers: trial of FUMEX questionnaire for assessing health problems in 125 French dock workers

David Lucas (1,2), Isabelle Despres (1), Dominique Jegaden (1,2), Brice Loddé (2,3)

Corresponding author:
David Lucas
(d.lucas@metrabrest.com)

(1) Occupational Health Service of Iroise, Brest, France
(2) French Society of Maritime Medicine, Brest, France
(3) Center of Occupational Health and Environmental Diseases Brest Teaching Hospital, Brest, France

**Background**

Four years ago, the European Society for Environmental and Occupational Medicine society published the FUMEX (Fumigation Exposure) questionnaire on the health impact of fumigants and other VOC's in containers. The aim of this study was to evaluate exposures and health impacts in dock workers.

**Methods**

Dock workers were interviewed face to face by a nurse or an occupational physician from the port occupational health center of Le Havre and Brest from April to September 2015. Questionnaires were analyzed by Sphinx Software.

**Results**

125 questionnaires were included and analyzed. Mean age was 35 years and 46% were smokers. More than 80% reported no exposures to fumigants or volatile organic compounds. The majority of exposures were reported by refrigeration technicians (21%) during port engine driving or when handling containers. Symptoms most frequently described were headaches (42%), fatigue (50%), sleeping disorders (33,6%), and pulmonary irritation (31%). Only 22.4% wore protective equipment regularly. Phosphine was the fumigant most frequently mentioned.

**Conclusions**

The dock workers who most frequently reported exposures in the questionnaire survey were refrigeration technicians and logistic workers. The lack of knowledge on risk and preventive measures was clear and was considered to be a major finding. The FUMEX questionnaire is too complicated to be relevant for daily prevention in occupational health services. It is more useful for physicians in hospital or researchers to address suspicious cases of exposure. Following this study, a shorter and more comprehensive questionnaire was created.

---

### Performance of specific immunoglobulin E tests for diagnosis of occupational asthma: a systematic review and meta-analysis

Harald Lux (1), Klaus Lenz (2), Xaver Baur (3)

Corresponding author:
Harald Lux
(harald.lux@charite.de)

(1) Charité – Universitätsmedizin Berlin, Institute of Occupational Medicine, Berlin, Germany
(2) Charité – Universitätsmedizin Berlin, Institute of Medical Biometrics and Clinical Epidemiology, Berlin, Germany
(3) European Society for Environmental and Occupational Medicine (EOM), Berlin, Germany

**Background**

Exposure to substances at worksites was estimated to cause more than 10% of new-onset asthma cases in adults.[1] International guidelines for occupational asthma (OA) recommend specific immunoglobulin E (sIgE) serum tests as part of diagnostic work-ups.[2] Since introduction, various sIgE test methods have been subject to research and have become partly commercially available. The aim of this study was to analyze the performance of sIgE tests for the diagnosis of OA. Primary objectives were test performances for high and low molecular weight, single or groups of antigens, and different test methods.

### Methods

This systematic review with meta-analysis was performed according to the PRISMA statement[3] and oriented on the Cochrane Handbook for DTA Reviews.[4] An electronic literature search based on pre-defined objectives and inclusion criteria was conducted in MEDLINE and EMBASE (covering 1967-2016). Initial results underwent screening, final inclusion, duplicate publication check, and data extraction procedures. Included studies underwent quality assessment with the QUADAS-2 tool. Data were analyzed in R.

### Results

Sixty-two of 71 studies included in the systematic review were quantitatively analyzed. Overall sensitivity for high (HMW) and low molecular weight (LMW) antigens was 0.77 [95% CI 0.71-0.82] and 0.34 [95% CI 0.23-0.46], for the group of wheat and rye 0.78 [95% CI 0.68-0.86] and for diisocyanates 0.27 [95% CI 0.19-0.39]. Overall summary receiver operating characteristic (SROC) curves for applicable data had an area under the curve (AUC) of 0.778 for HMW and 0.566 for LMW antigens. Patient selection and poor reporting notably affected the quality of studies. Sixteen studies had 15 or more eligible participants.

### Conclusions

The analysis confirmed and extended previous findings with respect to occupational asthma caused by HMW agents, which showed relatively high sensitivity of wide-ranging sIgE tests. Considerably lower overall sensitivity and heterogeneous results of LMW sIgE tests indicated methodological difficulties and/or different pathogenesis of LMW asthma (i.e. with partially non IgE driven asthma, e.g. caused by diisocyanates).

Some of the results had to be interpreted with caution because of limitations of the quality of included studies and number of participants.

### References

1. Kogevinas M, Zock JP, Jarvis D, Kromhout H, Lillienberg L, Plana E, et al. Exposure to substances in the workplace and new-onset asthma: an international prospective population-based study (ECRHS-II). Lancet [Internet] 2007 Jul 28 [cited 2017 Dec 4];370(9584):336-41. doi: 10.1016/S0140-6736(07)61164-7. Available from: http://www.thelancet.com/journals/lancet/article/PIIS0140-6736(07)61164-7/fulltext Subscription required to view
2. Aasen TB, Burge PS, Henneberger PK, et al. Diagnostic approach in cases with suspected work related asthma. J Occup Med Toxicol [Internet] 2013 Jun 14 [cited 2017 Dec 4];8(1):17. doi: 10.1186/1745-6673-8-17. Available from: https://occup-med.biomedcentral.com/track/pdf/10.1186/1745-6673-8-17?site=occup-med.biomedcentral.com
3. Liberati A, Altman DG, Tetzlaff J, Mulrow C, Gotzsche PC, Ioannidis JP, et al. The PRISMA statement for reporting systematic reviews and meta-analyses of studies that evaluate healthcare interventions: explanation and elaboration. BMJ Brit Med J [Internet] 2009 Jul 21 [cited 2017 Dec 4];339:b2700. doi: 10.1136/bmj.b2700. Available from: http://www.bmj.com/content/bmj/339/bmj.b2700.full.pdf

4. Deeks J, Wisniewski S, Davenport C. Chapter 4: Guide to the contents of a Cochrane Diagnostic Test Accuracy Protocol. In: Deeks J, Bossuyt P, Gatsonis C, Hrsg. Cochrane Handbook for Systematic Reviews of Diagnostic test Accuracy Version 100: The Cochrane Collaboration; 2013. Available from: http://methods.cochrane.org/sites/methods.cochrane.org.sdt/files/public/uploads/Ch04_Sep2013.pdf

### Risk communication as the important outcome of an epidemiological study: a case report

Jelena Macan, Željka Babi, Zrinka Frani

Corresponding author:
Jelena Macan
(jmacan@imi.hr)

(1) Institute for Medical Research and Occupational Health, Unit for Occupational and Environmental Health, Zagreb, Croatia

### Background

According to the World Health Organization, risk communication in public health includes strategies for encouraging informed decision making, positive behavior change and maintenance of trust with the aim to protect health. Adequate communication of occupational risk has a significant impact on the prevention of work-related diseases and preservation of workforce fitness. Risk communication is considered successful when the same specific risk is adequately perceived by both experts and the public, and communicated information is trustworthy.

### Methods

The present study presents risk communication strategies taken after the completion of an epidemiological study aimed at evaluation of work safety measures, prevalence and severity of skin symptoms and their impact on quality of life among Croatian hairdressing apprentices.[1]

### Results

Risk communication was performed on two levels: apprentices/their parents, and experts (teachers, physicians, safety experts, authorities in education and health). Communication at the apprentice level began with the provision of information about the aims of the study to potential study participants and their parents using informed consent forms and leaflets. Next steps involved an individual approach: information about evaluated risk factors provided at the time of examination, and individual medical reports including study results and advice. Communication at the expert level involved study reports for the school, lectures, materials for promotion of knowledge about risks and safe behavior, national guidelines for physicians, and participation in governmental working groups providing rules in the field of health at safety at work and education.

### Conclusions

Risk communication strategies have been more successful at the expert level, generating changes in school curriculum and medical practice, and interest in information about occupational health risks from vocational education authorities. Risk communication at the apprentice level seems more difficult due to individual differences in susceptibility to environmental hazards with poorly defined susceptibility markers and limited understanding of their contribution to risk levels.

### References

1. Samardzic T, Varnai VM, Bakotic M, Babic Z, Brans R, Cvijetić Avdagić S, et al. Skin health and safety at work in Croatian hairdressing apprentices. Contact Dermatitis [Internet] 2016 July [cited 2017 Dec 4];75(1):25-31. doi: 10.1111/cod.12603. Epub 2016 Apr 28. Available from: http://onlinelibrary.wiley.com/doi/10.1111/cod.12603/abstract Subscription required to view

---

### Health impacts related to air pollution: perspectives of the FUTURAR project

Ana Mendes (1,2), Solange Costa (1,2), Joana Ferreira (3), Carlos Silveira (3), Hélder Relvas (3), Myriam Lopes (3), Alexandra Monteiro (3), Peter Roebeling (3), Ana Isabel Miranda (3), João Paulo Teixeira (1,2)

Corresponding author:
Ana Mendes
(asestevao@gmail.com)

(1) Universidade do Porto, Instituto de Saúde Pública, EPIUnit (Epidemiology Research Unit), Porto, Portugal
(2) National Institute of Health, Environmental Health Department, Air and Occupational Health Unit, Porto, Portugal
(3) CESAM, Department of Environment and Planning, University of Aveiro, Portugal

### Background

Air pollutants cause a variety of adverse impacts on human health, including premature mortality and morbidity. Air pollution is an important stimulus for the development and exacerbation of respiratory diseases such as asthma, chronic obstructive pulmonary disease, and lung cancer, and makes a substantial impact on cardiovascular disease. Furthermore, air pollution accounts for 3.1% of global disability-adjusted life years (time spent in states of reduced health). Despite emissions decreases and air quality improvement in Europe and Portugal over the last decade, ozone and particulate matter atmospheric concentrations still exceed the legislated standards. In this context, the main goal of the FUTURAR project "*Air quality in Portugal in 2030 – a policy support*" is to assess environmental and health impacts, together with a cost-benefit analysis, associated with the air pollutants emission reductions for 2030 imposed by the National Emission Ceilings new Directive (NECD).

### Methods

FUTURAR addresses and overtakes policy-oriented research gaps, particularly country-specific exposure-response functions for many important morbidity endpoints using modelling tools to estimate the spatial distribution of environmental and health impacts in Portugal.

### Results
The reference exposure-response functions suitable for Portugal will be used to estimate the health impacts for each emission reduction scenario and produce reference maps for each selected health indicator. Health indicators include premature mortality from exposure to ozone ($O_3$) and particulate matter (PM) (Years of Life Lost and SOMO35 metrics), as well as additional relevant morbidity health indicators to be defined. In this context, this presentation focuses on the preliminary review and data collection on health effects and metrics related to the human exposure to air pollutants.

### Conclusions
This research supports public health policy strategies to be taken at the national and regional level by competent authorities to control the emissions of air pollutants using integrating assessment modelling for policy support of clean air strategies.

### Funding
This study is supported by FEDER through the Operational Program Competitiveness and Internationalization and national funding from the Foundation for Science and Technology – FCT (Portuguese Ministry of Science, Technology and Higher Education) (POCI-01-0145-FEDER-016752), under the project "FUTURAR - Air quality in Portugal in 2030: a policy support" (PTDC/AAG-MAA/2569/2014).

---

### Association between inherent and exposure-induced variation in DNA repair capacity and development of skin cancer among sun-exposed workers in Romania

Horatiu Moldovan (1), Minodora Dobreanu (1), William W. Au (1,2)

Corresponding author:
Horatiu Moldovan
(moldovan.horatiu@umftgm.ro)

(1) University of Medicine and Pharmacy of Tirgu-Mures, Romania;
(2) Shantou University Medical College, China

### Background
Carcinogenesis is a prolonged and multi-staged process, and DNA repair is a gateway mechanism which can determine whether cancer will occur. Since it is difficult to determine DNA repair capacity among normal individuals, our ability to predict risk for development of cancer is imprecise. On the other hand, there is an urgent need to develop useful assessment tools for workers exposed to carcinogens and to provide them with appropriate preventive and medical care.

### Methods
With the challenge Comet assay which was developed by Au, DNA repair capacity in response to UV-exposure can be quantitatively determined. In this project, sun-exposed workers and matched workers will be investigated. Blood lymphocytes from all workers will be exposed to UV-light in vitro and these cells will be “challenged” to repair the induced DNA damage. Repair capacity will be determined quantitatively using the Comet assay.

### Results

It is expected that the sun-exposed workers, on average, will have lower DNA repair capacity than the matched controls. Among both groups, those with greater sun-exposure (as determined by clinical parameters and UV exposure measurements) will have worse repair capacity than unexposed subjects. Among the case workers, those with lower repair capacity will be significantly associated with a history of skin cancer. Those with the worst capacity are expected to have the worst prognosis for their cancer.

### Conclusions

This innovative investigation can potentially be used to provide personalized risk assessment for the development and prognosis of cancer. Consequently, better prevention and intervention protocols can be developed.

---

### Cardiovascular assessment of employees with occupational exposure to carbon monoxide

Vlad-Ioan Morariu (1), Stelian-Ioan Morariu (2), Anca-Maria Popa (5), Doina Giurgiu (3), Horatiu Moldovan (4), Florica Pascu (1), Mirela Cleopatra Tomescu (1)

Corresponding author:
Vlad-Ioan Morariu
(v.i.morariu@gmail.com)

(1) Victor BabesćUniversity of Medicine and Pharmacy, Timisoara, Romania, Timisoara Insitiute of Cardiovascular Diseases
(2) VasileGoldisćWest University Arad, Occupational Health Department of the Arad County Clinical Emergency Hospital, Arad, Romania
(3) Lucian BlagaćUniversity of Medicine, Sibiu, Romania
(4) University of Medicine and Pharmacy of Tirgu-Mures, Romania
(5) “Louis Turcanu” Emergency Hospital for Children, Timisoara

### Background

Acute intoxication with carbon monoxide (CO) can have important cardiovascular consequences (arrhythmias, worsening of ischemic heart diseases through hypoxia).

However, the cardiovascular effects of chronic intoxication with CO is poorly documented, especially in the domain of occupational exposure (blast furnace workers, coke workers, foundry workers, petrol refinery workers, mechanics, welders, drivers, fireman, cooks, traffic workers, and chemical industry workers).

Hemoglobin, myoglobin and cytochrome p450 have a much greater affinity for CO than for $O_2$, leading to chronic tissue hypoxia (through carboxyhemoglobin), enhanced atherosclerosis (modified endothelial permeability and altered cholesterol deposition through p450 cytochrome). Studies performed by Sari et al[1,2] showed high levels of carboxyhemoglobin (due to occupational exposure) to be associated with increased levels of high specificity C reactive protein (hs-CRP) and advanced carotid atherosclerosis, as well as EKG modifications (related to potentially fatal arrhythmias).

### Objectives

The objective of the study is to analyze the association between chronic occupational exposure to CO and cardiovascular disorders in patients with no record of cardiovascular diseases prior to employment, as well as to assess the feasibility of certain cardiovascular screening methods in order to help improve prophylaxis of non communicable diseases that generate a high level of morbidity, mortality, as well as unemployment. With this aim, the present study focuses on a professionally active population group.

### Methods

The study will be a prospective epidemiological study, and inclusion criteria will include official chronic workplace exposure to CO, a minimum employment period of 5 years, age lower than 50 years, lack of cardiovascular comorbidities upon initial employment, and non-smoker status. Cardiovascular assessment will include blood pressure monitoring, ankle-brachial ratio measurement (a very useful atherosclerosis screening tool), vascular/carotid Doppler ultrasound, standard electrocardiogram, and blood tests (carboxy hemoglobin, hs-CRP, CK-Mb levels).

### Limitations

The main limitation is obtaining a pure exposure to CO as most subjects are passive smokers and are exposed to other fumes.

### References

1. Sari I, Zengin S, Ozer O, Davutoglu V, Yildirim C, Aksoy M. Chronic carbon monoxide exposure increases electrocardiographic P-wave and QT dispersion. Inhal Toxicol. 2008 Jul;20(9):879-84. doi: 10.1080/08958370801958622 . PubMed PMID:18645728.
2. Davutoglu V, Zengin S, Sari I, Yildirim C, Al B, Yuce M, Ercan S. Chronic carbon monoxide exposure is associated with the increases in carotid intima-media thickness and C-reactive protein level. Tohoku J Exp Med. 2009 Nov;219(3):201-6. PubMed PMID: 19851048.

### In vitro evaluation of the genotoxic properties of Pencycuron, a commonly used phenylurea fungicide, by the cytokinesis-block micronucleus assay

Karoly Nagy (1), Congying Zheng (1), Balazs Adam (1), Claudia Bolognesi (2)

Corresponding author:
Karoly Nagy
(nagy.karoly@sph.unideb.hu)

(1) University of Debrecen, Faculty of Public Health, Department of Preventive Medicine, Debrecen, Hungary
(2) Ospedale Policlinico San Martino, National Cancer Institute, San Martino-IST Environmental Carcinogenesis Unit, IRCCS Genoa, Italy

### Background

Pencycuron, a phenylurea-type antifungal agent, is used in agriculture worldwide for inhibiting the growth of various fungal pathogens that invade rice, cotton, potato and several seedlings. In Europe, it has been found at concentrations close to 0.01 mg/kg in potato and lettuce, 0.02 mg/kg in soils, and 0.005 mg/kg in drinking water. Accordingly, human occupational and consumer exposure can be expected to be significant. A recent study has shown that pencycuron exerts genotoxic effects in zebrafish during early stages of development. Our yet unpublished preliminary research utilizing the comet assay has demonstrated dose-dependent DNA damage induced by pencycuron in *in vitro* models of human peripheral blood lymphocytes and human hepatocytes (HepG2). A statistically significant genotoxic effect was observed at 10 μg/ml concentration in lymphocytes as measured by tail DNA% ($p=0.040$), and at 100 μg/ml in hepatocytes as measured by tail length ($p=0.037$) and tail moment ($p=0.023$). Nevertheless, published human toxicity studies on its carcinogenic, mutagenic or genotoxic potential are lacking. Therefore, our aim is to confirm whether pencycuron exposure can lead to increased DNA damage in human cells investigated by the cytokinesis-block micronucleus (CBMN) assay in collaboration with the Environmental Carcinogenesis Unit Ospedale Policlinico San Martino IRCCS, Genova, Italy.

### Methods

The laboratory technique applicable to this collaboration is the CBMN assay, which is a comprehensive approach for evaluating genomic damage in different cell lines and cultured peripheral lymphocytes. The CBMN test involves the block of cytokinesis with cytochalasin B (Cyt-B) and allows the evaluation of the micronucleus frequency in once divided cells accumulated in the binucleated stage.

### Conclusions

The results of the CBMN assay can be correlated to the results of the comet assay and this comprehensive information can help in assessing health risk and drawing conclusions regarding recommended preventive measures to avoid unnecessary exposure and negative health consequences.

### Future impact of climate and precursor emission change on ozone-related mortality in Europe

Hans Orru (1,2), Camilla Andersson (3), Tanel Tamm (4), Kristie L. Ebi (1), Bertil Forsberg (1)

Corresponding author:
Hans Orru
(Hans.Orru@ut.ee)

(1) Umea University, Department of Public Health and Clinical Medicine, Umea, Sweden
(2) University of Tartu, Department of Family Medicine and Public Health, Tartu, Estonia
(3) Swedish Meteorological and Hydrological Institute, Norrköping, Sweden
(4) University of Tartu, Institute of Ecology and Earth Sciences, Tartu, Estonia

### Background
Ozone is a highly oxidative pollutant and is associated with increased mortality. All else (e.g. ozone precursors) being equal, summertime ground-level ozone will increase as temperatures increase with climate change. As a part of the 'Assessment of changing conditions, environmental policies, time-activities, exposure and disease' (ACCEPTED) project we used greenhouse gas and ozone precursor emission scenarios, global and regional climate and chemistry-transport models, epidemiological data and population projections to assess ozone-related health risks under a changing climate.

### Methods
European ozone concentrations were modelled at a grid size of 50 x 50 km using Multi-scale Atmospheric Transport and Chemistry MATCH along climate projections from the regional climate model RCA4. For the surface ozone, the global climate model EC-Earth was used as input for the regional climate model, forced by the greenhouse gas emission scenario RCP4.5. The climate during 1991-2000 was compared with the future climate from 2046–2055. The impacts on long- and short-term mortality due to ozone exposure-related premature deaths in Europe were assessed.

### Results
Currently, long-term exposure to ozone causes around 55,000 premature deaths annually in Europe, of which around 26,000 deaths are expected due to short-term effects. Taking into account only the impact of changing climate on surface ozone, up to an 11% increase in ozone-associated mortality is expected in some countries in central and southern Europe. However, a projected decrease in ozone precursor emissions will cause a much larger decrease in surface ozone (29.5% of EU average). Due to aging and increasingly susceptible populations, the decrease in 2050 would be actually smaller, up to 24.2%. During the summer months, ozone risks will combine with heat hazards, especially during the hottest periods and in densely populated urban areas.

### Conclusions
Near-surface ozone has significant effects on health. Although it is projected to be smaller in the future (mainly due to precursor emission decreases), the reduction is not as large as it could be due climate change and population increases.

### Health impacts of oil shale sector air pollution in eastern Estonia

Hans Orru (1,2), Jelena Tomasova (3), Jane Idavain (1), Kaja Julge (4), Jüri Ruut (1), Leena Albrecht (3), Kaisa Kesanurm (5), Kristina Aidla (3), Hedi Harzia (3)

Corresponding author:
Hans Orru
(Hans.Orru@ut.ee)

(1) University of Tartu, Department of Family Medicine and Public Health, Tartu, Estonia
(2) Umea University of Tartu, Department of Public Health and Clinical Medicine, Umea, Sweden
(3) Health Board, Tallinn, Estonia
(4) University of Tartu, Institute of Clinical Medicine, Tartu, Estonia
(5) Estonian Environmental Research Centre, Tallinn, Estonia

### Background

Oil shale is a mineral resource that is used for electricity generation, as well as producing shale oil, phenols, and other chemical products. The study aimed to identify oil shale processing impacts on the residents' health in eastern Estonia.

### Methods

The air pollution exposure assessment used the measured and modelled oil shale sectors' induced concentrations of benzene, formaldehyde, phenol and PM2.5. The epidemiological "Study of the health impact of oil shale sector – SOHOS" consisted of questionnaires among adults and children, spirometry and exhaled NO data among 9-10 yrs. old schoolchildren (n=1000) and the ecological analysis used cancer registry data. The results in eastern Estonia were compared with Tartu as the control area.

### Results

Over half of the residents in Ida-Viru county reported being worried about air quality. They complained more frequently ($p<0.05$) about tightness, long-term cough, phlegm in the lungs, wheezing and heart diseases, hypertension, stroke, diabetes and stenocardia. People living in regions with higher levels of benzene, phenol, formaldehyde or PM2.5 had significantly higher odds ($p<0.05$) of experiencing shortness of breath, asthma attacks, long-term cough, phlegm in the lungs, wheezing or chest tightness, as well as heart attacks or angina during the past year.

Children in Ida-Viru county had significantly more ($p<0.05$) dry cough, mucous secretion and allergic rhinitis compared to children living in Tartu. The prevalence of asthma is higher than in any previous study in Estonia. In some schools, up to 15% of children had high values of exhaled nitrogen oxide, but no asthma diagnosis.

The cancer registry analysis showed that men living in municipalities of oil shale mining in Ida-Viru county had higher lung cancer incidence rates during an 18-year study period compared to the average incidence rate for the rest of Estonia.

### Conclusions

People living in eastern Estonia have more health complaints and diseases related to, among other things, environmental pollution. Currently, additional studies have been initiated to identify children's exposures and health effects in greater detail.

---

### Role of polycyclic aromatic hydrocarbons in the biological effects from combustion-derived particulate matter

Johan Øvrevik (1), Bendik C. Brinchmann (1), Tonje S. Skuland (1), Alena Kubátová (2), Klára Kukowski (2), Jana Rousová (2), Eric le Ferrec (3), Dominique Lagadic-Gossmann (3), Marit Låg (1), Magne Refsnes (1), Jørn Holme (1), Per Schwarze (1)

Corresponding author:
Johan Øvrevik
(johan.ovrevikl@fhi.no)

(1) Norwegian Institute of Public Health, Oslo, Norway
(2) University of North Dakota, Department of Chemistry, Grand Forks, ND, USA
(3) Inserm U1085, Institut de Recherche en Santé, Environnement, Travail, Rennes, France & Université de Rennes 1, Faculté des Sciences pharmaceutiques et biologiques, Rennes, France

### Background

A central topic of the current research at the Dept. of Air Pollution and Noise, NIPH, is the importance of chemical composition and specific sources on the health effects from air pollution. Polycyclic aromatic hydrocarbons (PAHs) are among the main drivers of carcinogenesis from exposure to combustion-derived particulate matter (CPM) such as diesel exhaust (DEP) and wood-smoke particles (WSP). However, the role of PAHs in other adverse outcomes remains elusive. Currently, we are investigating their potential role in inflammation responses and intracellular calcium signaling.

### Methods

Organic compounds were extracted from DEP and WSP according to polarity and chemically characterized. Extracted fractions were tested for cytotoxicity and pro-inflammatory effects in human lung epithelial cells lines (BEAS-2B) and for pro-inflammatory effects and calcium signaling in endothelial cells (HMEC-1 and primary human endothelial cells).

### Results

DEP, but not WSP, triggered pro-inflammatory responses through lipophilic compounds. PAH-content did not correlate with these effects, but PAH-rich extracts of DEP and WSP exacerbated pro-inflammatory responses induced through Toll-like receptor 3 (TLR3). Similarly, pyrene, a dominating PAH in DEP, WSP and PM2.5, but not benzo[a]pyrene (B[a]P), also enhanced TLR3-induced expression of pro-inflammatory genes. Compared to BEAS-2B cells, gene-expression in endothelial cells were triggered at lower concentrations, with primary endothelial cells being considerably more sensitive. Lipophilic soluble compounds were the main drivers of effects, and the responses were accompanied by increased intracellular calcium. Comparable calcium-responses could also be triggered by pyrene and B[a]P.

### Conclusions

The results show that different constituents may contribute to the biological effects of combustion particles. PAHs do not appear to be the main drivers of inflammatory responses, but could exacerbate activation of pro-inflammatory responses by other agents and induce calcium signaling, potentially affecting a number of cellular functions. The study also underscores the need clarify effects from PAHs other than B[a]P.

---

### Particle emissions from residential biomass combustion—emission levels and relevant health characteristics

Joakim Pagels (1), Axel Eriksson (1), Christina Andersen (1), Birgitta Svenningsson (2), Robert Lindgren (3), Christoffer Boman (3)

Corresponding author:
Joakim Pagels
(Joakim.Pagels@design.lth.se)

(1) Lund University, Ergonomics & Aerosol Technology, Lund, Sweden
(2) Lund University, Department of Physics, Lund, Sweden
(3) Umeå University, Department of Applied Physics & Electronics, Umeå, Sweden

### Background

Exposure to emissions from combustion sources in the form of traffic and residential wood combustion have been linked to adverse health effects in the population. Particle emissions from biomass combustion are responsible for an increasing share of the PM exposure in Europe and have been estimated to cause premature mortality of at least 40.000 deaths per year in Europe.[1] Residential fuel combustion (biomass and coal) is also the dominant source of genotoxic compounds such as polycyclic aromatic hydrocarbons (PAHs) in European air.[2]

### Methods

Particle emissions were investigated from common wood log and wood pellet stoves. Additionally, these emissions are undergoing simulated atmospheric processing to investigate formation of additional secondary particle mass in the atmosphere. Emissions were diluted and measured with high time-resolution using aerosol mass spectrometry to allow us to identify the combustion conditions responsible for elevated emissions.

### Results

Wood log stove emissions are dominated by solid black carbon (BC) particles and emission factors were found to range from 800-2000 mg/kg fuel. Specific combustion conditions were identified where emissions of genotoxic compounds such as PAHs were increased by up to two orders of magnitude.[3] This included use of large batches of very dry fuel in conventional wood stoves. Atmospheric transformation adds more organic mass to the particles and changes their properties. Using modern wood pellet stoves dramatically decreases emissions of solid black carbon particles, organic components and PAHs. Differences in particle composition and emission levels between residential biomass combustion and traffic will be discussed.

### Conclusions

Residential biomass combustion is a major source of ambient particle concentrations and emission levels can be reduced by modern technologies. In addition, particle composition varies strongly with combustion conditions, which has important implications for health effects in the population. The identified combustion conditions leading to strongly elevated PAH emissions may be an explanation for the dominance of residential biomass combustion as a source of PAHs in European air.

### References

1. Sigsgaard T, Forsberg B, Annesi-Maesano I, Blomberg A, Bolling A, Boman C, et al. Health impacts of anthropogenic biomass burning in the developed world. Eur Respir J [Internet] 2015 Dec [cited 2017 Dec 4]:46(6):1577-88. doi: 10.1183/13993003.01865-2014. Epub 2015 Sep 24. Available from: http://erj.ersjournals.com/content/erj/46/6/1577.full.pdf
2. European Environmental Agency. Air Quality in Europe – 2014 Report. Luxembourg. 2014. Report number 5. doi: 10.2800/22775. Available from: https://www.eea.europa.eu/publications/air-quality-in-europe-2014
3. Nielsen IE, Eriksson AC, Lindgren R, Martinsson J, Nyström R, Nordin EZ, et al. Time-resolved analysis of particle emissions from residential biomass combustion – Emissions of refractory black carbon, PAHs and organic tracers. Atmos Environ [Internet] 2017 Sep [cited 2017 Dec 4];165:179-90. doi: 10.1016/j.atmosenv.2017.06.033. Available from: https://ac.els-cdn.com/S1352231017304144/1-s2.0-S1352231017304144-main.pdf?_tid=0b9292e2-d911-11e7-801d-00000aab0f02&acdnat=1512405469_d8a74c8c84724de76a3dd2c0f599d087

### Funding

This project was financed by the Swedish Research Councils VR and FORMAS.

---

### Climate change and public health: assessment of the effects of extreme environmental conditions and development of innovative prevention and mitigation strategies

Anastasia K. Paschalidou (1), Pavlos Kassomenos (2), Haritini Tsangari (3), Clare Heaviside (4), Sotiris Vardoulakis (5), Kyriakos Georgiou (6), Edna N. Yamasaki (6)

Corresponding author:
Anastasia K. Paschalidou
(apascha@fmenr.duth.gr)

(1) Democritus University of Thrace, Department of Forestry and Management of the Environment and Natural Resources, Orestiada, Greece
(2) University of Ioannina, Department of Physics, Laboratory of Meteorology, Ioannina, Greece

(3) University of Nicosia, Department of Economics and Finance, Nicosia, Cyprus
(4) Chemical and Environmental Effects Department, Public Health England, Oxon, United Kingdom
(5) Institute of Occupational Medicine, Edinburgh, United Kingdom
(6) University of Nicosia, Nicosia, Cyprus

### Background

There is an almost unanimous scientific consensus that climate change is the biggest environmental problem facing the world. Existing evidence indicates that thermal stress related to climate change is leading to an increase in heat-related deaths as well as an increase in the number of cardiovascular, cerebrovascular and respiratory incidents globally. In a typical Mediterranean environment like Cyprus, where severe Saharan dust-storms are very common, the adverse health effects of extreme weather and particulate air pollution are expected to be substantial.

### Methods

The CYPHEW project studies the impact of extreme summer weather and particulate pollution on mortality and morbidity in Cyprus for current and future climate scenarios and develops a heat-health-watch-warning (HHWW) system for the protection of public health in urban areas in Cyprus. This should facilitate the improvement of quality of life and the enhanced management of health services.

### Results

Implementation of a generalized linear model with quasi-Poisson regression revealed the exposure-response function for heat mortality in Cyprus, and identified a temperature threshold of 33.7°C, above which mortality risk increased sharply. Specifically, for a 1°C increase in temperature above the threshold, the exposure-response coefficient was 1.042, reflecting an approximate rise in mortality of 4.2%. The model was extended to examine the confounding effect of air pollution.[1] Additionally, we investigated the relationship between synoptic weather types and cardiovascular and respiratory hospital admissions. We found that morbidity correlates with the occurrence of cold, rainy days and high humidity levels.[2] A health impact assessment revealed 32 heat-related deaths per year in Cyprus, while the potential future heat-related mortality (when including climate projections) was estimated to double for a 1°C increase in temperature and to reach 800% for a 5°C increase, in line with expected estimates by the end of the century.[3]

### Conclusions

This work highlights the importance of predictability through HHWW systems and the impact of extreme environmental conditions on public health, particularly with a changing climate.

### References

1. Tsangari H, Paschalidou A, Kassomenos AP, Vardoulakis S, Heaviside C, Georgiou KE, et al. 2015b. Extreme weather and air pollution effects on cardiovascular and respiratory hospital admissions in Cyprus. Sci Total Environ [Internet] 2016 Jan 15 [cited 2017 Dec 4];542(PtA):247-53. doi: 10.1016/j.scitotenv.2015.10.106. Epub 2015 Oct 28. Available from: http://www.sciencedirect.com/science/article/pii/S0048969715309220?via%3Dihub Subscription required to view

2. Tsangari H, Paschalidou A, Vardoulakis S, Heaviside C, Konsoula Z, Christou S, et al. Human mortality in Cyprus: the role of temperature and particulate air pollution. Reg Environ Chang [Internet] 2015 [cited 2017 Dec 4];16(7):1905-13. Available from: https://link.springer.com/article/10.1007/s10113-015-0793-2 Subscription required to view
3. Heaviside C, Tsangari H, Paschalidou A, Vardoulakis S, Kassomenos P, Georgiou KE, et al. Heat-related mortality in Cyprus for current and future climate scenarios. Sci Total Environ [Internet] 2016 Nov [cited 2017 Dec 4];569-570:627-33. doi: 10.1016/j.scitotenv.2016.06.138. Epub 2016 Jul 1. Available from: http://www.sciencedirect.com/science/article/pii/S0048969716313079?via%3Dihub Subscription required to view

### Acknowledgements
This work was co-funded by the European Regional Development Fund and the Republic of Cyprus through the Research Promotion Foundation (Project ΥΓΕΙΑ/ΔΥΓΕΙΑ/0609(ΒΙΕ)/20)

---

### Micro solid phase extraction: a green technique for determination and biomonitoring of endocrine disruptor organophosphates

**Fusun Okçu Pelit (1), Levent Pelit (1), Ilknur Bagatir Erbas (1), Tugba Yavuz (1), Tugberk Nail Dizdas (1), Tulin Deniz Ciftci (1), Kasim Ocakoglu (2)**

Corresponding author:
Fusun Okçu Pelit
(fusun.okcu@ege.edu.tr)

(1) Ege University Translational Pulmonology Research Group (EgeTPAG). Ege University, Faculty of Science, Department of Chemistry, Izmir, Turkey
(2) Mersin University, Advanced Technology, Research and Application Center Mersin, Turkey

### Background
Recent reports have identified chemicals capable of disrupting the endocrine system in environmental samples called endocrine disrupting compounds (EDCs). Organophosphate EDCs have been linked to ecological impacts at trace concentrations and detected in various samples. Therefore, there is an urgent need to develop sensitive methods to be able to detect concentrations of these chemicals at an ng $L^{-1}$ level. To reach this level, residue analysis is carried out in sequence pre-concentration steps which are tedious, time consuming and labor intensive. In this study, we present a cheap, fast, environmentally safe sample preparation procedure which will improve the quality of analytical results in a greener way.

### Methods
Solid phase extraction (SPE) has been employed for pre-concentration of pesticides as a standard method. However, this method has some drawbacks such as multistage operation, large consumption of reagents, a low enrichment factor, and is time consuming. These problems can be overcome by using the micro-SPE (μ-SPE) technique. In this work, information on a novel green extraction methodological approach including μ-SPE is presented. Different methods, including dispersive, magnetite and micropipette tipped types were used for fast analysis of EDC pesticides in water samples. Different nano particles, namely, $TiO_2$, ZnO, $SiO_2$, a novel magnetic $Fe_3O_4$/Ni/NixB nanocomposite, and different clay types were utilized in fast micro extraction of pesticides. These nanofiller surfaces were used in dispersive µ-SPE, in micropipette tipped extraction and the magnetic µ-SPE procedure.

### Results

The parameters related to extraction efficiency were screened. The performance of the extraction of EDC pesticides was compared using gas chromatographic systems. The regression coefficients were at least 0.99. Recoveries from spiked well waters range from 80 to 97% and RSDs were no higher than 15%.

### Conclusions

These micro extraction methods share the advantages of classical extraction methods, including excellent enrichment performance, easy operation and the ability to employ a wide range of "green" extraction techniques and hence they can be used in the analysis of clinical specimens during the exposure phase.

### Mercapturates of cysteine-S-disulfides for risk assessment of exposure-related hypertension and kidney dysfunction

Clara G Dias, Nuno R Coelho, Maria J Correia, Patrícia C Gracio, Lucília N Diogo, Emília C Monteiro, Sofia A Pereira

Corresponding author:
Sofia A. Pereira
(sofia.pereira@nms.unl.pt)

(1) CEDOC, Chronic Diseases Research Center, NOVA Medical School | Faculdade de Ciências Médicas, Universidade NOVA de Lisboa, Lisboa, Portugal

### Background

Blood pressure regulation and the mercapturate pathway are interlinked and mostly dependent on the kidney.[1] The end-products of this pathway are mercapturates generated upon cysteine-S-conjugates acetylation by N-acetyltransferase 8 (NAT8) activity. Coenzyme A (CoA) is a reaction's co-product.[2] We aimed at biomonitoring the NAT8 products from normotension to hypertension.

### Methods

An animal model of hypertension induced by chronic intermittent hypoxia (CIH) was used. In this model, blood pressure increases progressively and hypertension is observable after 2 weeks.[3]
We obtained the longitudinal data for elimination products of cysteine-S-conjugates (mercapturates (uNAC) and CoA at day 0, 1, 7 and 14 of CIH-exposure. A control group submitted to normoxic conditions was used. uNAC and CoA were quantified by HPLC-FD.[4]

### Results

There was no variation of uNAC or uCoA throughout the study in control. There was a two-fold increase in uNAC upon CIH-exposition at d1 and d7, which decreased until d14 ($p < 0.001$; 2-way-ANOVA). This profile was not observed for CoA, and elimination was maintained without differences between groups (2-way-ANOVA).

### Conclusions

Data showed a growing generation of cysteine-S-disulfides at the kidney with blood pressure increase. When hypertension is established, the kidney seems to fail mercapturate generation. This data supports the biomonitoring of mercapturates of cysteine-S-disulfides for risk assessment of exposure-related kidney dysfunction and hypertension.

### References

1. Juhanson P, Kepp K, Org E, Veldre G, Kelgo P, Rosenberg M, et al. N-acetyltransferase 8, a positional candidate for blood pressure and renal regulation: resequencing, association and in silico study. BMC Med Genet [Internet] 2008 Apr 10 [cited 2017 Dec 4];9:25. doi: 10.1186/1471-2350-9-25. Available from: https://www.ncbi.nlm.nih.gov/pmc/articles/PMC2330028/pdf/1471-2350-9-25.pdf
2. Veiga-da-Cunha M, Tyteca D, Stroobant V, Courtoy PJ, Opperdoes FR, Van Schaftingen E. Molecular Identification of NAT8 as the Enzyme That Acetylates Cysteine S-Conjugates to Mercapturic Acids. J Biol Chem [Internet] 2010 Jun 11 [cited 2017 Dec 4];285(24):18888-98. doi: 10.1074/jbc.M110.110924. Epub 2010 Apr 13. Available from: https://www.ncbi.nlm.nih.gov/pmc/articles/PMC2881811/pdf/zbc18888.pdf
3. Diogo LN, Pereira SA, Nunes AR, Afonso RA, Santos AI, Monteiro EC. Efficacy of carvedilol in reversing hypertension induced by chronic intermittent hypoxia in rats. Eur J Pharmacol [Internet] 2015 Oct 15 [cited 2017 Dec 4];765:58-67. doi: 10.1016/j.ejphar.2015.08.019. Epub 2015 Aug 18. Available from: http://www.sciencedirect.com/science/article/pii/S001429991530193X?via%3Dihub Subscription required to view
4. Dias CG, Diogo LN, Campos P, Lemos AR, Monteiro EC, Karina Soto, et al. Urinary N-acetyl cysteine-disulfides and kidney disease progression in HIV-infected patients: a prospective study. J Am Soc Nephrol 2016; 27:952-3A.

### Funding

Fundação para a Ciência e Tecnologia, iNOVA4Health - UID/Multi/04462/2013 (Ref: 201601-02-021); PhD grants for CGD, NRC and MJR

### DNA methylation and autoantibody biomarker development strategies for minimally invasive diagnostics

Walter Pulverer, Klemens Vierlinger, Christa Noehammer, Andreas Weinhaeusel

Corresponding author:
Walter Pulverer
(Walter.Pulverer@ait.ac.at)

(1) AIT – Austrian Institute of Technology, Vienna, Austria

### Background

There is a great need for and anticipation of the identification of novel biomarkers for different types of diseases at the earliest possible stages, which can ideally be integrated in minimally invasive diagnostic assays. DNA methylation changes and autoantibodies are able to detect the onset of cancer months or years before its clinical diagnosis and can therefore be used as biomarkers for early diagnosis.

### Methods and Results

In recent years, we have developed and optimized epigenome- and immunome-wide discovery and validation technologies for the identification of novel biomarkers. From microarray-based discovery studies we have defined specific multivariate classifiers for different diseases (e.g. cancer, ulcerative colitis, etc.) with almost perfect diagnostic performance, obtaining AUC-values of up to 0.9-1 for the big 4 cancer entities. Efficient targeted multiplexed technologies were established for validation of the findings from the discovery studies. Analysis based on methylation sensitive restriction digests coupled with micro fluid high-throughput qPCR is able to detect 0.1-1% of methylated DNA in a limited amount (10 ng) of cell free DNA in plasma. As proof of principle we have tested 680 plasma samples, and were able to obtain AUC values of 0.84-0.91 for the 4 different lung cancer subtypes using a multiplexed methylation test. Additionally, we have tested these samples using autoantibody profiles. Therefore, we developed a 100-plex Luminex assay for testing candidate antigenic markers and conducted multimodal analyses combining both autoantibody and DNA methylation data for improving cancer diagnostics. Moreover, recent developments from protein-based towards peptide-based tools for autoantibody and immunome analyses done on breast cancer showed improved diagnostic performance on our customized 179k peptide-array.

### Conclusions

Based on our findings in different (cancer) studies, both DNA methylation- and autoantibody-based strategies surpass the current clinical diagnostic methods and could be of value to improve both disease diagnosis and patient management.

---

### Promoting health in small and artisanal mining of gold (PROSAMIGO): feasibility study for human biological monitoring of mercury exposure

Ilse Ottenbros (1), Romilda Z. Boerleider (1), Bianca Jubitana (2), Jan Quik (3), Nel Roeleveld (1), Paul T. J. Scheepers (1)

Corresponding author:
Paul T. J. Scheepers
(paul.scheepers@radboudumc.nl)

(1) Radboud University Medical Center, Radboud Institute for Health Sciences, Radboudumc, Nijmegen, The Netherlands
(2) Medische Zending Primary Health Care Suriname, Paramaribo, Suriname;
(3) Centraal Laboratorium, Bureau voor Openbare Gezondheidszorg, Paramaribo, Suriname

### Background

The use of mercury in artisanal and small-scale gold mining (ASGM) has consequences for human health and the environment. We conducted the PROmoting health in Small and Artisanal MIning of GOld (PROSAMIGO) study to explore the situation for mercury and health in the gold mining areas in the inland of Suriname, South America. The aim of our project was to study the feasibility of introducing a human biological monitoring (HBM) program to assess mercury exposure of gold miners and local residents, including children.

### Methods

A literature eview was performed to determine the most suitable biological media for HBM. We also interviewed 30 gold miners and 76 residents to determine their interest to participate in an HBM program and included questions asking what they could do to reduce the uptake of mercury from the environment.

### Results

The literature study resulted in factsheets to support application of HBM to assess exposure of metallic and methyl mercury. We prepared a diagram that can support decision-making regarding the most suitable biological medium for HBM (blood, urine, hair or exhaled air, see: http://www.aimspress.com/article/10.3934/environsci.2017.2.251). Local staff interviewed both villagers and gold miners in their native language. The respondents expressed an interest in knowing their mercury body burdens and gave their consent to collect specimens, from themselves and their children as well. Only a few respondents did not want to provide samples for HBM.

### Conclusions

We consider it feasible to prepare an HBM program on the inland of Suriname. For the successful introduction of HBM, it is important to carefully report lab results to each participant, together with information on possible solutions and adequate care, tailored to each inidividual's situation.

### Funding acknowledgement

PROSAMIGO received funding from the Uitvoeringsorganisatie Twinning Suriname Nederland (UTSN) of the Dutch Ministry of Foreign Affairs.

---

### How surface charge changes the toxicokinetics of nanoparticles

Paul T. J. Scheepers, Arné Oerlemans, Jorien Sanderink, Ilse Ottenbros

Corresponding author:
Paul T. J. Scheepers
(paul.scheepers@radboudumc.nl)

(1) Radboud University Medical Center, Radboud Institute for Health Sciences, Radboudumc, Nijmegen, The Netherlands

### Background

Nanoparticles (NPs) with a negative or positive surface charge (anions or cations) demonstrate a distinct interaction with biological tissues compared to their non-charged counterparts. This work aims to integrate and analyze current knowledge of charge properties of NPs on the molecular, cellular and organism level.

### Methods

In a search in Pubmed and Medline performed from May-June 2014, we used the terms ‘surface charge’, ‘charged particle’, ‘charged molecule’, ‘anion’, ‘cation’, ‘air ionization’, ‘ionized air’, and ‘air ions’. These search terms were combined with ‘toxicokinetics’, ‘health effects’, ‘reproduction’ and ‘neoplasms’. Non-English publications and papers on mass spectrometry or radiation were excluded. This resulted in 1,051 articles.

### Results

In a biological medium, the interaction of charged NPs with tissues depends on how a charged surface is coated. Hydration does not necessarily affect the charge properties of NPs, but increases their hydrodynamic diameter. Lipids and proteins reduce NP charge. These NPs interact with cell membranes that have an overall negative charge. Electrostatic interaction results in accumulation of cationic NPs and explains their rapid internalization. Inside the cells, charged NPs interact with the endoplasmic reticulum, mitochondria and DNA, ultimately leading to toxicity and apoptosis. Anionic NPs trigger the response of phagocytizing cells leading to endocytosis. In non-phagocytizing cells these NPs depend on localized positively charged patches in the phospholipid bilayer for their cellular uptake. Inhaled anionic NPs are rapidly translocated from alveoli to the blood circulation, whereas cationic NP escape from clearance, showing more retention, accumulation and toxicity in epithelial cells. The influence of charge on the uptake via other routes (gastrointestinal or skin) indicate that similar mechanisms apply as in the airways.

### Conclusions

The results from *in vitro*, animal and very limited human data indicate that there is consistent evidence to suggest that charge properties of NPs have a profound influence on their kinetics and toxicity.

### Application of systematic review methodology to risk factors of non-communicable disease in the field of occupational health

Daniele Mandrioli (1), Vivi Schlünssen (2,3), Stevie van der Mierden (1), Daria Sgargi (4), Paul T. J. Scheepers (5)

Corresponding author:
Paul T. J. Scheepers
(paul.scheepers@radboudumc.nl)

(1) Ramazzini Institute, Cesare Maltoni Cancer Research Centre, Bentivoglio, Bologna, Italy

(2) Aarhus University, Department of Public Health, Section for Environment, Occupation and Health, Danish Ramazzini Centre, Aarhus, Denmark
(3) National Research Center for the Working Environment, Copenhagen, Denmark
(4) Department of Statistical Sciences, University of Bologna, Italy
(5) Radboud University Medical Center, Radboud Institute for Health Sciences, Radboudumc, Nijmegen, The Netherlands

**Background**

Protecting workers' health requires a framework of workplace standards for exposures and exposure levels that is well-informed by science. Robust methods to evaluate available scientific evidence to reach conclusions regarding the strength of evidence are fundamental to facilitate the required translation of scientific knowledge into workplace standards. In clinical sciences, methods of evidence integration and systematic reviews have played a transformative role in the timely incorporation of scientific insights into clinical practice. Since 2009, researchers have begun to explore the application of systematic and robust methods of evidence integration in the environmental health sciences. To date, the Navigation Guide has been the most applied systematic review (SR) methodology in environmental health. This experience is now available to be used in the field of occupational health.

**Methods**

In the DiMoPEx project, we have organized workshops, webinars and a training school to learn about systematic review methodology and the authors have participated in a webinar on the Navigation Guide. During two short-term scientific missions that have recently begun, we plan to further explore the value of SR methodology in occupational health using pneumoconiosis as a case study.

**Results**

During a workshop (Copenhagen, April 2017) and training school (Debrecen, June 2017) we discussed the Navigation Guide, PRISMA, COHSTER and GRADE. We also discussed the value of pre-publication of SR protocols. Currently, we are working on the application of SR methods to the case of pneumoconiosis.

**Conclusions**

The present study considers the application of systematic review methods in environmental health for non-communicable disease risk factors in the field of occupational health.

**Acknowledgements**

DiMoPEx experts who participated in the workshop and training school. We would also like to acknowledge Paul Whaley of the University of Lancaster, Lancaster, UK for his role as instructor in the training school in Debrecen, as well as Tracey Woodruff and Juleen Lam of the University of California San Francisco for a webinar they provided on the Navigation Guide.

### Nitrate in drinking water and colorectal cancer

Jörg Schullehner (1,2,3), Birgitte Hansen (1), Malene Thygesen (2), Carsten Bøcker Pedersen (2), Torben Sigsgaard (3)

Corresponding author:
Torben Sigsgaard
(ts@mil.au.dk)

(1) Geological Survey of Denmark and Greenland, Aarhus, Denmark
(2) National Centre for Register-Based Research, School of Business and Social Sciences, Aarhus University, Denmark
(3) Aarhus University, Department of Public Health, Section for Environment, Occupation & Health, & Danish Ramazzini Centre, Aarhus, Denmark

### Background

Nitrate in drinking water is suspected to be a cause of colorectal cancer due to endogenous transformation into carcinogenic N-nitroso compounds. Epidemiological evidence is limited, and previous studies were often challenged by estimating long-term exposure on an individual level.

### Methods

We exploited longitudinal population-based data on health and drinking water quality to study the impact of nitrate in drinking water on colorectal cancer risk. We calculated nitrate exposures for the entire Danish population, based on water quality analyses in public water supply areas and private wells between 1978 and 2011. Follow-up started at age 35. We used Cox proportional hazards models to estimate hazard ratios (HRs) of nitrate exposure quintiles and a trend-summary statistic adjusting for potential confounders.

### Results

Results from this PhD project under the dNmark research alliance will be presented. In short, we found a positive association between nitrate in drinking water and colon cancer risk for females and for both sexes combined. However, no statistically significant association was found for males only. An increased hazard was found for the highest exposed quintiles for females and for both sexes combined below the current drinking water standard.

### Conclusions

Our results add to the body of evidence suggesting an increased risk of colon cancer at nitrate concentrations in drinking water well below the current drinking water standard. A discussion of the adequacy of drinking water standards with regard to chronic effects is warranted.

### Atmospheric ammonia, ammonium and incident asthma: nationwide case-control study in Danish preschool children

Gitte Holst (1), Malene Thygesen (2), Carsten B. Pedersen (2), Robert G. Peel (3), Jørgen Brandt (3), Jesper H. Christensen (3), Jakob Bønløkke (1), Ole Hertel (3)*, Torben Sigsgaard (1)*

*Ole Hertel and Torben Sigsgaard share the final author position.

Corresponding author:
Torben Sigsgaard
(ts@mil.au.dk)

(1) Aarhus University, Deparment of Public Health, Section for Environment, Occupation & Health, & Danish Ramazzini Centre, Aarhus, Denmark
(2) National Centre for Register-Based Research, School of Business and Social Sciences, Aarhus University, Denmark
(3) Department of Environmental Science, Aarhus University, Roskilde, Denmark

### Background
Particulate matter less than 2.5 μm in diameter ($PM_{2.5}$) has repeatedly been associated with respiratory illness. The role of particulate constituents such as ammonium is, however, less clear.

### Objective
We investigated gaseous ammonia ($NH_3$), particulate ammonium ($NH_{4+}$), the total concentration of these pollutants ($NH_x$), and total $PM_{2.5}$ and their association with asthma in Danish preschool children.

### Methods
We used a register-based matched case-control design. Cases comprised children with first diagnosis of asthma from general practices or hospitals (n=12,948) from their 1st to their 6th birthday during 2006-2012. Per case, we selected 25 controls at random (n=323,700) having no asthma diagnosis and matched on sex and birthday. Modeled average concentrations of $NH_3$, $NH_{4+}$, $NH_x$ and $PM_{2.5}$ (5.6 km x 5.6 km grid resolution) during the last 3, 6 and 12 months prior to diagnosis were linked to registry data on residential coordinates, asthma diagnosis and covariate information.

### Results
There was a positive association between $NH_3$ exposure (adjusted hazard ratio (($HR_{adj}$, 95%CI) 1.74, 1.6-1.90), $NH_{4+}$ exposure ((HRadj, 95%CI) 2.33, 2.012.6), $NH_x$ exposure (($HR_{adj}$, 95%CI) 1.88, 1.69-2.09) and cases of asthma (3-month exposure time window). The direction of these associations changed when adjusting for region and socio-economic status, but remained when $NH_{4+}$ was adjusted for total $PM_{2.5}$. $PM_{2.5}$ exposure was not associated with asthma ($HR_{adj}$, 95%CI) 0.96, 0.86-1.1). Similar results were obtained for 6- and 12-month exposure time windows.

### Conclusions

$NH_3$ and $NH_{4+}$ exposure may be risk factors of onset asthma in preschool children. Further prospective exploration in large-scale populations is needed to confirm the results and foreclose confounding.

### Acknowledgments

Funding organizations dNmark and NordicWelfAir (NordForsk under the Nordic Programme on Health and Welfare).

### Genetic effects of *in utero* exposure to tobacco smoke: the NeoGENE project

Carla Costa (1,2), Ana Teresa Reis (1,2), Ana Inês Silva (1,2), Filipa Esteves (1,2), Sérgio Barros (1), Alla Tirsina (1), Fernando Barbosa Jr. (3), João Paulo Teixeira (1,2)

Corresponding author:
João Paulo Teixeira
(joao.teixeira@insa.min-saude.pt, jpft12@gmail.com)

(1) National Institute of Health, Environmental Health Department, Air and Occupational Health Unit, Porto, Portugal
(2) EPIUnit - Instituto de Saúde Pública, Universidade do Porto, Porto, Portugal
(3) School of Pharmaceutical Sciences of Ribeirão Preto, University of São Paulo, Ribeirão Preto, Brazil

### Background

In addition to smoking during pregnancy, environmental tobacco smoke (ETS) exposure should also be considered when discussing the effects of tobacco, as tobacco smoke has been classified by the USEPA as a class A carcinogen. In the last few years, it has been shown that *in utero* exposures to maternal smoking may be associated with both genetic and epigenetic effects. It remains uncertain if in utero exposure to ETS may have similar effects. Moreover, most studies do not consider possible co-exposure to other relevant chemicals, and therefore there is a critical need for more complete environmental exposure data in these studies.

### Methods

The present study aims to assess the damage in DNA and changes in patterns of DNA methylation and histone modifications resulting from *in utero* exposure to ETS (confirmed with analysis of cotinine concentrations). The study population will include pregnant women and newborns of the Porto district. In order to address simultaneous exposure to other DNA-damaging chemicals, a wide number of chemicals will also be analyzed in biological matrices, including PAHs, phthalates and metals.

### Results

Data obtained will include not only genetic damage levels and DNA methylation alterations, but also information on other possible relevant co-exposures and their smoking status. Altogether, results will contribute to deepening the knowledge of the risk associated with maternal exposure to ETS.

### Conclusions

Information collected can provide scientific support for the possible implementation of coherent and effective health promotion measures in order to optimize the current and future health not only of women, but also their children.

### Acknowledgments

This work is supported by FCT and FAPESP (FAPESP/19914/2014); Carla Costa is supported by FCT (SFRH/BPD/96196/2013).

### Optimizing urine-derived cell staining for the human micronucleus assay

Filipa Esteves (1,2), Raquel Amaro (1,2), Susana Silva (1), Solange Costa (1,2,3), Carla Costa (1,2), Claudia Bolognesi (4), João Paulo Teixeira (1,2)

Corresponding author:
João Paulo Teixeira
(joao.teixeira@insa.min-saude.pt, jpft12@gmail.com)

(1) National Institute of Health, Environmental Health Department, Air and Occupational Health Unit, Porto, Portugal
(2) EPIUnit - Instituto de Saúde Pública, Universidade do Porto, Porto, Portugal
(3) Universidade do Porto, Instituto de Ciências, Tecnologias e Agroambiente (ICETA), Porto, Portugal
(4) Ospedale Policlinico San Martino, National Cancer Institute, San Martino-IST Environmental Carcinogenesis Unit, IRCCS Genoa, Italy

### Background

Micronucleus formation has been widely used in toxicology as a biomarker of chromosomal damage, genomic instability and carcinogenic events. Most cancers are of epithelial origin and therefore the micronucleus test (MN) in urine derived cells (UDC) is of a great importance. Furthermore, these cells are collected via a minimally invasive procedure, an important advantage for human biomonitoring studies. The main problem regarding UDC is the lack of standardization of MN protocol, and staining in particular. Current data show a variety of methods which may lead to bias, and giemsa and feulgen are the most commonly used stains. Giemsa staining allows quick preparation of slides for microscopic evaluation. However, the distinction between micronucleus and other cellular structures/nuclear abnormalities is not easily accomplished, since as a non-DNA-specific stain it may favor false positive readings. On the other hand, Feulgen staining is DNA-specific and allows for good contrast between nucleus and cytoplasm. The use of different stains and protocols may contribute to the large inter-laboratory variations and inconsistencies found in studies.

### Methods

The present study aims to apply different staining techniques in UDC from human samples and stablish a detailed set of criteria for scoring all of the biomarkers of UDC. Urine samples will be collected from a group of individuals. Urine derived cells will be isolated and fixed. Giemsa staining and Feulgen staining technique will be performed for each sample. Reading will be performed using both light and fluorescence microscopy.

### Conclusions

The results of this study will be used to standardize the application of the MN assay in UDC, through selection of the most reliable UDC staining method and establishment of scoring criteria.

### Funding Acknowledgements

This work was supported by Project NORTE-01-0145-FEDER-000010 - Health, Comfort and Energy in the Built Environment (HEBE), cofinanced by Programa Operacional Regional do Norte (NORTE2020), through Fundo Europeu de Desenvolvimento Regional (FEDER)

---

### Human exposure to noise and development of cardiovascular disorders: an epigenetic analysis

João Paulo Teixeira (1,2), Carlos Carvalhais (3), Paula Neves (1,2), Blanca Laffon (3), Cristiana Costa Pereira (1,2)

Corresponding author:
João Paulo Teixeira
(joao.teixeira@insa.min-saude.pt, jpft12@gmail.com)

(1) National Institute of Health, Environmental Health Department, Air and Occupational Health Unit, Porto, Portugal
(2) EPIUnit - Instituto de Saúde Pública, Universidade do Porto, Porto, Portugal
(3) Universidade da Coruña, DICOMOSA Group, Department of Psychology, Area of Psychobiology, A Coruña, Spain

### Background

Noise is a major confounding factor in many polluted living and working environments.[1] Recently the World Health Organization established noise as the second largest environmental cause of health problems, just after the impact of air quality.[2]

Long-term exposure to relevant noise levels has been shown to be associated with negative health outcomes,[3] and evidence from epidemiological studies demonstrates that environmental noise is associated with an increased incidence of arterial hypertension, myocardial infarction, and stroke. A growing body of research also suggests that noise can impact cardiovascular risk, from disrupting night-time sleep and raising stress levels to increasing blood pressure and heart rate, and reducing vascular function.[4]

Epigenetic alterations are potentially major mechanisms by which environmental factors can affect physiological functions and disease risk. Epigenetic mechanisms include DNA methylation and microRNA alterations that can be recognized and used as diagnostic or prognostic markers. Some of these epigenetic alterations have already been described for cardiovascular disease in environmental exposure experiments.

### Methods

This study has been designed to enroll 180 subjects exposed to noise, occupationally and non-occupationally, in six different settings, and 60 subjects apparently non-exposed to noise, as the control population. It will consist of two fundamental phases: the first includes a thorough description of the exposure scenarios with measurements and broad-spectrum analysis of noise levels and the study population, which will be comprehensively characterized through questionnaires on noise exposure, quality of life and cardiovascular disease risk through intensive field work. The second phase will consist of epigenetic testing for cardiovascular disease markers and evaluation of DNA damage levels in all subjects.

### Conclusions

The results obtained will be analyzed for statistical associations, and risk ratios will be established. This study is expected to bring advances to relevant areas such as noise impact, epigenetic markers use, and public health data, with benefits to both workers and the general population.

### References

1. Eurostat. Work and health in the EU: A statistical portrait. Office for Official Publications of the European Communities; 2004.
2. World Health Organisation (WHO). Burden of disease from environmental noise. Copenhagen: WHO Regional Office for Europe; 2011. ISBN: 978 92 890 0229 5. Available from: http://www.euro.who.int/__data/assets/pdf_file/0008/136466/e94888.pdf
3. Gourevitch B, Edeline J-M, Occelli F, Eggermont JJ. Is the din really harmless? Long-term effects of non-traumatic noise on the adult auditory system. Nat Rev Neurosci [Internet] 2014 Jul [cited 2017 Dec 5];15(7):483-91. doi: 10.1038/nrn3744. Available from: https://www.nature.com/articles/nrn3744 Subscription required to view
4. Passchier-Vermeer W, Passchier WF. Noise exposure and public health. Environ Health Persp [Internet] 2000 Mar [cited 2017 Dec 5];108(Suppl 1):123-31. Available from: https://www.ncbi.nlm.nih.gov/pmc/articles/PMC1637786/pdf/envhper00310-0128.pdf

### Constructing a job exposure matrix for lung carcinogens

Nur Z. Toreyin (1), Ozlem Goksel (2), Tuncay Goksel (2), Suriye Ozgur (3), Lode Godderis (4,5)

Corresponding author:
Nur Z. Toreyin
(zehra.nur.toreyin@ege.edu.tr, ntreyin@gmail.com)

(1) Ege TPAG. Ege University, Department of Public Health, Division of Work Health and Occupational Diseases, Izmir, Turkey
(2) Translational Pulmonology Research Group (EgeTPAG), Ege University, Faculty of Medicine, Department of

Pulmonary Medicine, Division of Asthma, Immunology and Allergy, Izmir, Turkey
(3) EgeTPAG. Ege University, Faculty of Medicine, Ege University, Department of Biostatistics and Medical Informatics, Izmir, Turkey
(4) Centre for Environment and Health, Department of Public Health and Primary Care, Leuven, Belgium
(5) IDEWE - External Service for Prevention and Protection at Work, Heverlee, Belgium

### Background

The identification of occupational carcinogens and the causal relationship between occupational exposure and cancer remain difficult.[1] A detailed exposure history is a basic requirement to link a clinical cancer with occupation.[2] The main aim of this project is to develop a quantitative job exposure matrix (JEM) to be used in epidemiologic studies on occupational lung cancer in Belgium. The secondary outcome involves adapting it for use with a Turkish working population.

### Methods

Job exposure matrices are cross-tabulations of jobs and exposures by which exposures in occupational settings can be estimated. Exposure estimates are derived from combinations of occupation, activity and period for each agent. Each cell provides information on probability and level of exposure.[3] Estimates can be made in a qualitative (yes-no) or a quantitative (low-intermediate-high) manner.[3,4,5] Agents of interest were selected from group 1, group 2A and group 2B of the IARC (International Agency for Research on Cancer) classification as of December 2016. Historical biomonitoring datasets that were accessed through the largest Belgian Occupational safety and Health (OSH) service as well as exposure data of Belgian OSH services reported to Belgian authorities were interpreted for quantitative estimates. Occupations (ISCO-08) and economic activities (NACE) generated the occupational dimension. The time dimension covered the period 1998-2017.

### Results

A total of 43139 samples was selected for 10 known, 2 probable, and 6 possible carcinogens between 1998 and 2017. These agents will be used in the final model. There were 228 and 251 unique ISCO-08 and unique NACE codes corresponding to each biomonitoring result, respectively. 1-hydroxypyreen, aluminum, arsenic, cadmium, chromium and nickel level in urine samples were above the upper reference limits (URL) for the Belgian adult population, but below occupational exposure limits.

### Conclusions

Statistical models will be developed to predict probability and level of exposure in further analyses. The final model will be adapted to lung cancer patients in Belgium.

### References

1. Doll R, Peto R. The causes of cancer: quantitative estimates of avoidable risks of cancer in the United States today. JNCI - J Natl Cancer I [Internet] 1981 Jun [cited 2017 Dec 5];66(6):1192-308. Available from: https://academic.oup.com/jnci/article-abstract/66/6/1192/1076736?redirectedFrom=fulltext Subscription required to view
2. Takala J. Eliminating occupational cancer. Ind Health [Internet] 2015;53(4):307-9. doi: 10.2486/indhealth.53–307. Available from: https://www.ncbi.nlm.nih.gov/pmc/articles/PMC4551060/pdf/indhealth-53-307.pdf
3. Kauppinen T, Toikkanen J, Pukkala E. From cross tabulations to multipurpose exposure information systems: a new job exposure matrix. Am J Ind Med [Internet] 1998 Apr [cited 2017 Dec 5];33(4):409-17. Available from: http://onlinelibrary.wiley.com/doi/10.1002/(SICI)1097-0274(199804)33:4%3C409::AID-AJIM12%3E3.0.CO;2-2/abstract Subscription required to view

4. Peters S, Vermeulen R, Portengen L, Olsson A, Kendzia B, Vincent R, et al. SYN-JEM: A Quantitative Job-Exposure Matrix for Five Lung Carcinogens. Ann Occup Hyg [Internet] 2016 Aug [cited 2017 Dec 5];60(7):795-811. doi: 10.1093/annhyg/mew034. Epub 2016 Jun 9. Available from: https://watermark.silverchair.com/mew034.pdf?token=AQECAHi208BE49Ooan9kkhW_Ercy7Dm3ZL_9Cf3qfKAc485ysgAAAaowggGmBgkqhkiG9w0BBwagggGXMIIBkwIBADCCAYwGCSqGSIb3DQEHATAeBglghkgBZQMEAS4wEQQMZrWH7RbmBnNnjHnrAgEQgIIBXT-8WTV0NMI2cIuz06q3yLZrYOB72g2JogYLeKeDNWsLThZv9M0uG1q66Qu_DEQjfg8HLhWMtCjGgbssvMKsNKHMq6kZbFptcR7_7NHtEhegfkmM7508BlRkAXSyg0cjCeX5sKHZL-GgBo5dXrE9YI0FLc9VjX2Do6yEZSt6c39ZGYhPfpR_Bp-jZdoqs_MGucoZUALwPQK9gTqXOWpZViLsGNn21bBQPeIF1weWB62IGa8_JdWLcnw7KzcX4hQHVWJfx_UkzENmlrzgrR0XAQv6mBlnbqyszGmXppC0ph6Q10PY0RWXzlmsS0y0n5pxDUF1uNye6eCe4iiotXfueJOkUqwq7DbtXhh-LfGgxtOqxvjoEf_63H6_a-wc70pzjjTmNmKAr9J6j6dvpKUzo6g3NCk_Ryho9WNUoezFCg4wRzVTWcTP5YmhNx_X11tNEgIXJZnOtD9xmOcBxTk

5. Févotte J, Dananché B, Delabre L, Ducamp S, Garras L, Houot M, et al. Matgéné: a program to develop job-exposure matrices in the general population in france. Ann Occup Hyg [Internet] 2011 Oct [cited 2017 Dec 5];55(8):865-78. doi: 10.1093/annhyg/mer067. Epub 2011 Sep 15. Available from: https://watermark.silverchair.com/mer067.pdf?token=AQECAHi208BE49Ooan9kkhW_Ercy7Dm3ZL_9Cf3qfKAc485ysgAAAakwggGlBgkqhkiG9w0BBwagggGWMIIBkgIBADCCAYsGCSqGSIb3DQEHATAeBglghkgBZQMEAS4wEQQM0-5M_

### Toxicity assessment of iron oxide nanoparticles for biomedical applications

Natalia Fernández-Bertólez (1), Carla Costa (2,3), Fátima Brandao (2,3), Sónia Fraga (2,3), João Paulo Teixeira (2,3), Eduardo Pásaro (1), Blanca Laffon (1), Vanessa Valdiglesias (1,3)

Corresponding author:
Vanessa Valdiglesias
(vvaldiglesias@udc.es)

(1) Universidade da Coruña, DICOMOSA Group, Department of Psychology, Area of Psychobiology, A Coruña, Spain
(2) National Institute of Health, Environmental Health Department, Porto, Portugal
(3) Universidade do Porto, Instituto de Saúde Pública, EPIUnit (Epidemiology Research Unit), Porto, Portugal

### Background

Due to their unique physicochemical properties, iron oxide nanoparticles (IONs) offer promising possibilities for biomedical applications, including diagnosis and drug delivery. Previous studies showed that ION can cross intact blood-brain barriers. Although in general, IONs show good biocompatibility, their accumulation and effects on nervous cells need to be comprehensively clarified. The ION surface is frequently coated with a variety of materials in order to avoid aggregation, to increase their stability, and to offer a platform for functionalization. Little is known of how these coatings may influence ION interaction with biological structures. Therefore, the present project aims to determine the possible toxicity at the cellular and molecular level of differently coated IONs, initially focusing on their effects on nervous system cells.

### Methods

After physicochemical characterization of the ION, a complete set of assays will be applied to determine cytotoxicity, genotoxicity and effects on DNA repair mechanisms induced by exposure of neuronal and glial cells to IONs. Experimental conditions will involve short- and long-term incubations, several doses, and presence/absence of protein-rich serum in the cell culture media.

### Results

In view of the extensive current use and promising future applications of IONs, especially those which involve direct administration in the human body, it is necessary to fully understand the interactions of these nanomaterials with cellular systems, as well as their potential adverse health consequences.

### Conclusions

Results obtained in this work will shed light on the possible cellular and molecular effects of IONs, which will help to define conditions for using these nanomaterials under minimal reasonable risks to human health, thus guaranteeing manufacturers' and consumers' safety.

### Acknowledgments

Funded by Xunta de Galicia (grant numbers ED431B 2016/013 and ED481B 2016/190-0) and the project NanoToxClass (ERA-SIINN/001/2013). F. Brandão is supported by the grant SFRH/BD/101060/2014, funded by FCT (subsidized by national fund of MCTES).

---

### Plasma levels of TIMP-1 in patients with chronic obstructive pulmonary disease

Dimo Dimov (1), Tanya Tacheva (2), Cvetoslava Christozova (2,3), Tatyana Vlaykova (1)

Corresponding author:
Tatyana Vlaykova
(tvlaykov@mf.uni-sz.bg)

(1) Department of Internal Medicine, Medical Faculty, Trakia University, Stara Zagora, Bulgaria
(2) Department of Chemistry and Biochemistry, Medical Faculty, Trakia University, Stara Zagora, Bulgaria
(3) Undergraduate student

### Background

The characteristic features of the pathogenic mechanisms of chronic obstructive pulmonary disease (COPD) are abnormal chronic inflammation in the airways and extensive tissue remodeling. Among the different types of factors implicated in lung tissue remodeling are matrix metalloproteinases (MMPs) and their specific tissue inhibitors - TIMPs. The aim of the current study was to elucidate the possible role of plasma level of TIMP-1 as biomarker of COPD.

### Methods

We carried out the present analysis using ELISA method TIMP-1 plasma levels in 59 patients and 20 controls.

### Results

Plasma TIMP-1 levels were significantly higher in patients than in controls (3.94±2.84 vs. 1.78±0.62 ng/ml, p=0.001, Mann-Whitney test). The receiver operating characteristics (ROC) curve produced area under the curve (AUC) of 0.742 with 95%CI of 0.636-0.849 (p=0.001). The values above 1.70 ng/ml determined COPD with a sensitivity of 0.78, but with a low specificity of 0.50 (p=0.017, $\chi 2$ test). The levels of TIMP-1 did not differ significantly between non-smokers and ex/current smokers, but there was a tendency for a positive association with the smoking index of the latter (packs/year, Rho=0.282, p=0.061).

The TIMP-1 levels showed a weak positive correlation with the age of disease onset (Rho=0.260, p=0.050) and a weak negative correlation (Rho=-0.301, p=0.024) with the period from the disease diagnosis. In addition, TIMP-1 levels were significantly higher in patients with COPD GOLD II stage (4.38±2.51 ng/ml) than those with GOLD III (3.56±3.16 ng/ml, p=0.044, Mann-Whitney test), but not higher than those with GOLD IV (4.06±2.49 ng/ml, p=0.758)

### Conclusions

Our results suggest that the plasma TIMP-1 is increased in patients with COPD and is associated with some clinical parameters and cigarette use in smokers, but more evidence is needed before considering it to be a valuable biomarker for this disease.

### Funding sources

This work was supported by project 4/2016 of MF, TrU, Bulgaria and COST Action CA15129 (DiMoPEx).

---

### IL6 -174G>C promoter polymorphism may influence progression of cutaneous malignant melanoma

Tatyana Vlaykova (1), Mateuzh Kurzawski (2), Tanya Tacheva (1), Dimo Dimov (3), Asen Anastasov (1), Yoana Georgieva (1,4), Petya Peeva (5), Marek Drozdzik (2)

Corresponding author:
Tatyana Vlaykova (tvlaykov@mf.uni-sz.bg)

(1) Department of Chemistry and Biochemistry, Medical Faculty, Trakia University, Stara Zagora, Bulgaria
(2) Department of Experimental and Clinical Pathology, Pomeranian Medical University, Szczecin, Poland
(3) Department of Internal Medicine, Medical Faculty, Trakia University, Stara Zagora, Bulgaria
(4) Undergraduate student
(5) Division of Dermatology and Oncology, Oncology Center, Stara Zagora, Bulgaria

### Background

Cutaneous malignant melanoma (CMM) is the most life-threatening skin neoplasia and exhibits peculiar behavior; it is extremely aggressive with a rapid fatal outcome and spontaneous regression. Melanoma is one of the most immunogenic cancers and occurs 6-times more frequently than any others. These facts, as well as its responsiveness to different immunotherapies suggest the important role of the host immune system for the development, progression and outcome of this neoplasia.
In the current study we aimed to explore the possible role of two functional promoter polymorphisms in the pro-inflammatory cytokenes IL-6 and TNF-a in CMM (IL6 -174G>C, rs1800785 and TNFA -308G>A, rs1800629).

### Methods

We genotyped 76 patients with CMM and 198 non-affected control individuals by polymerase chain reaction - restriction fragment length polymorphism (PCR-RFLP) methods.

### Results

The genotype frequencies for both IL6 -174G>C and TNFA -308G>A SNPs did not differ significantly between patients and controls (p=0.810 and p=0.358). There were no significant associations between the genotypes and clinical and biochemical tumor characteristics. Carriers of the IL6 -174GG genotype tended to have shorter survival than the carriers of C allele genotypes (GC+CC) (mean 132.58 vs. 166.55 mo, p=0.299). After stratification for gender, this difference between genotype carriers appeared stronger (p=0.082; 182.44 vs. 229.90 mo in women and 46.01 vs. 98.57 mo in men). Similarly, after stratification for occupational conditions (harmful/non-harmful) the difference became highly significant (p=0.005; 185.73 vs. 256.00 mo in non-harmful and 24.09 vs. 104.33 mo in harmful).

### Conclusions

Based on our results, we could suggest that the promoter polymorphisms -174G>C in IL6 and -308G>A in TNFA are not predisposing factors for CMM, while the IL6 -174G>C SNP is likely to influence the progression of the disease, as the GG genotype may determine an unfavorable prognosis.

### Funding sources

This work was supported by project 3/2015 of MF, TrU, Bulgaria and COST Action CA15129 (DiMoPEx).

### Differences in particle chemical composition around an occupied residence

Yuliya Omelekhina (1), Axel Eriksson (1,2), Patrik Nilsson (1), Joakim Pagels (1), Aneta Wierzbicka (1)

Corresponding author:
Aneta Wierzbicka
(aneta.wierzbicka@design.lth.se)

(1) Lund University, Ergonomics and Aerosol Technology, Lund, Sweden
(2) Nuclear Physics, Lund University, Lund, Sweden

### Background

People in developed countries spend about 65% of their time in private homes,[1] underscoring the importance of understanding the consequences of residential particle exposures, yet knowledge is sparse. Indoor particulate matter (PM) comes from indoor sources and infiltrate from outdoors, can form from gas-phase precursors emitted both indoors and outdoors, and undergoes various dynamic transformations in confined indoor spaces. In our study, we aimed to investigate the differences in aerosol chemical composition inside and outside of an occupied residence.

### Methods

Simultaneous measurements were performed in a naturally ventilated Swedish residence in a multi-family house during a one-month period in the winter time. A time-of-flight aerosol mass spectrometer (HR-ToF-AMS) was used for quantification of size-resolved mass concentrations (PM1) of non-refractory species, coupled to an automatic switching valve alternating between the indoor and outdoor sampling position.[2]

### Results

Total average particle mass concentrations were higher inside (12.8 μg/m$^3$) than outside (5.3 μg/m$^3$) of the residence. Indoor to outdoor (I/O) ratios were 6.7 for organic aerosol, 0.5 for sulphate, 0.2 for nitrate, 0.2 for ammonium, 0.2 for chlorine. Indoor PM exposures were dominated by organic aerosol from indoor sources. The high organic I/O ratio is mainly explained by a high contribution from indoor sources and a low air exchange rate, giving a long life-time of indoor emissions. The high I/O ratio means penetration of organic matter from ambient pollution showed only a minor contribution to indoor exposures of organic PM. The results for non-volatile sulphate and chlorine reflected reduced infiltration from outdoors. Ammonium nitrate losses were observed upon moving from a colder outdoor to a warmer indoor environment, due to its sensitivity to changes in temperature and relative humidity.

### Conclusions

The study showed clear differences in exposure levels and PM1 particle chemical composition between indoors and outdoors. Aerosol levels indoors were mainly influenced by indoor sources, and these should not be neglected when considering possible health effects.

### References

1. Brasche S, Bischof W. Daily time spent indoors in German homes--baseline data for the assessment of indoor exposure of German occupants. Int J Hyg Envir Heal [Internet] 2005 [cited 2017 Dec 5];208(4):247-53. doi: 10.1016/j.ijheh.2005.03.003. Available from: http://www.sciencedirect.com/science/article/pii/S1438463905000635?via%3Dihub Subscription required to view
2. DeCarlo PF, Kimmel JR, Trimborn N, Northway MJ, Jayne JT, Aiken AC, et al. Field-deployable, high-resolution, time-of-flight aerosol mass spectrometer. Anal chem [Internet] 2006 Dec 15 [cited 2017 Dec 5];78(24):8281-9. doi: 10.1021/ac061249n. Available from: http://pubs.acs.org/doi/abs/10.1021/ac061249n Subscription required to view

### Funding acknowledgement

Funding from the Swedish Research Council Formas is greatly acknowledged.
